# Characterizing the Cattle Gut Microbiome in Farms with a High and Low Prevalence of Shiga Toxin Producing *Escherichia coli*

**DOI:** 10.3390/microorganisms9081737

**Published:** 2021-08-14

**Authors:** Karla Vasco, Brian Nohomovich, Pallavi Singh, Cristina Venegas-Vargas, Rebekah E. Mosci, Steven Rust, Paul Bartlett, Bo Norby, Daniel Grooms, Lixin Zhang, Shannon D. Manning

**Affiliations:** 1 Department of Microbiology and Molecular Genetics, Michigan State University, East Lansing, MI 48824, USA; vascokar@msu.edu (K.V.); nohomovi@msu.edu (B.N.); psingh1@niu.edu (P.S.); sloupreb@msu.edu (R.E.M.); lxzhang@msu.edu (L.Z.); 2 Department of Large Animal Clinical Sciences, College Veterinary Medicine, Michigan State University, East Lansing, MI 48824, USA; mcvv82@gmail.com (C.V.-V.); bartle16@msu.edu (P.B.); norby@msu.edu (B.N.); dgrooms@iastate.edu (D.G.); 3 Department of Animal Science, Michigan State University, East Lansing, MI 48824, USA; rust@msu.edu; 4 Department of Epidemiology and Biostatistics, Michigan State University, East Lansing, MI 48824, USA

**Keywords:** microbiota, cattle, Shiga toxin producing *Escherichia coli*, bacterial shedding

## Abstract

Cattle are the main reservoirs of Shiga toxin producing *Escherichia coli* (STEC), a major foodborne pathogen associated with acute enteric disease and hemolytic–uremic syndrome in humans. A total of 397 beef and dairy cattle from 5 farms were included in this study, of which 660 samples were collected for 16S rRNA gene sequencing. The microbiota of farms with a high-STEC prevalence (HSP) had greater richness compared to those of farms with a low-STEC prevalence (LSP). Longitudinal analyses showed STEC-shedders from LSP farms had higher microbiome diversity; meanwhile, changes in the microbiome composition in HSP farms were independent of the STEC shedding status. Most of the bacterial genera associated with STEC shedding in dairy farms were also correlated with differences in the percentage of forage in diet and risk factors of STEC carriage such as days in milk, number of lactations, and warm temperatures. Identifying factors that alter the gut microbiota and enable STEC colonization in livestock could lead to novel strategies to prevent fecal shedding and the subsequent transmission to humans.

## 1. Introduction

Shiga toxin producing *Escherichia coli* (STEC) is a foodborne pathogen causing 2.8 million cases of acute enteric disease and 230 deaths annually [1]. STEC infections are associated with the consumption of contaminated food and water or result from direct contact with cattle feces since cattle represent an important reservoir for this pathogen [2]. While livestock carriers of STEC are asymptomatic, humans can develop bloody diarrhea, hemolytic–uremic syndrome, thrombotic thrombocytopenic purpura, or end-stage renal disease [1]. STEC virulence is caused by bacteriophage-encoded Shiga toxins (Stx1 and Stx2) that induce cellular apoptosis of endothelial cells in the gut, kidney, and brain of humans [3,4,5,6]. Cattle are more tolerant to STEC due to the lack of Stx receptors (glycolipid globotriaosylceramide, Gb3) in the intestinal tract as well as a lower receptivity of Gb3 receptors present in the kidney and brain [7]. Some STEC strains, classified as enterohemorrhagic *E. coli* (EHEC), possess the locus of enterocyte effacement (LEE) pathogenicity island that encodes for a type III secretion system and effectors, such as the intimin protein (*eae*), which are responsible for attaching and effacing (AE) lesion formation [8]. Adult cattle carrying EHEC are typically unaffected, though infected calves develop AE lesions on the apical epithelial surfaces of the recto-anal junction where the bacteria colonize [9].

Because cattle are important reservoirs of STEC, reducing carriage of this pathogen in livestock and preventing dissemination in food and the environment are priorities for preventing human infections [10]. Our previous study identified risk factors associated with high STEC prevalence in dairy farms including first lactation, less than 30 days in milk, and warm temperatures [11]. Meanwhile, protective factors identified in farms with low STEC prevalence included access to pasture, anthelmintic treatment, and antibiotic treatment for respiratory infections [11]. Nonetheless, it is not clear how factors associated with STEC prevalence influence the microbiota composition and potentially favor STEC colonization.

The gut microbiome is critical for the activation and regulation of the immune response and for preventing pathogen colonization [12]. Some studies have analyzed the association between the gut microbiome and STEC in both humans and cattle. In humans, the gut microbiome of infected patients had a lower abundance of dominant taxa from Bifidobacteriales and Clostridiales [13]. We also previously showed that microbial communities from patients with acute enteric infections caused by STEC and other pathogens had a lower bacterial richness with an increased abundance of Proteobacteria (genus *Escherichia*) and decreased abundance of Bacteroidetes compared to healthy communities [14,15]. With regard to cattle, varying results have been observed in the richness and composition of the fecal microbiome between STEC shedders and non-shedders [16,17,18,19,20,21,22,23,24]. Within a specific farm, for example, some studies observed no difference in diversity among STEC shedders and non-shedders [19,21], whereas other studies detected significantly higher [22] and lower [23] diversity in STEC shedders despite controlling for age, farm, and diet. This lack of consensus among previous reports compels further investigation.

Herein, we sought to compare the microbiota structure and function of cattle among farms with a high versus low STEC prevalence. Additionally, we aimed to determine whether STEC carriage is associated with changes in the microbiota composition over time. Characterizing a healthy cattle microbiome that does not support pathogen colonization and identifying key beneficial microorganisms can guide the development of new prevention protocols to eradicate STEC colonization in animal reservoirs.

## 2. Materials and Methods

### 2.1. Sample Collection

An initial study was carried out in the spring and summer of 2011 and 2012 in Michigan in which 1096 samples were collected in 11 cattle farms including 6 dairy herds and 5 beef herds to determine STEC prevalence and identify risk factors for shedding [11]. Here, samples from 5 of those 11 cattle herds were selected for microbiome analysis, which was based on the varying prevalence of STEC in each herd. Specifically, a low STEC prevalence (LSP) was observed in one feedlot, 1B (8.2%), and two dairy farms, 2D (8.7%) and 4D (13.8%). Comparatively, the prevalence was considerably higher in feedlot 8B and dairy farm 9D (53.7% and 28.0%, respectively), which were classified as herds with a high STEC prevalence (HSP). The three dairy herds had Holstein cows (farms 2D, 4D, 9D), while the other two were beef feedlots with Crossbreed (farm 1B) and Angus (farm 8B) breeds. Epidemiological information obtained from each herd included demographics, geographic location, husbandry practices, health management, and diet. Additional information including number of lactations, days in milk, and dry status was collected at the dairy farms.

Fecal grabs (FGs) were collected by rectal palpation using obstetric sleeves (*n* = 308), while recto-anal junction (RAJ) samples were collected by swabbing the RAJ with a sterile cotton swab (*n* = 352) as described [11]. Roughly 256 pairs of fecal grabs (FGs) and rectal-anal junction (RAJ) swabs were collected simultaneously from the same animal for microbiome comparison. A subset of cattle was also sampled over time at an interval of 2 to 3 weeks between each sampling point to examine microbiome changes and STEC shedding over time (Table S1). In addition, blood samples were collected from the coccygeal or jugular vein of each animal for serology [25]. 

### 2.2. Pathogen Identification

STEC was detected using CHROMagar STEC and sorbitol MacConkey agar followed by PCR confirmation targeting key virulence genes [11]. Suspect isolates were classified as STEC if they were positive for any Shiga toxin gene (*stx*) subtype with or without the intimin gene (*eae*). In addition, exposure to pathogens that can alter the gut microbiota was evaluated to account for confounding effects between STEC shedders and non-shedders. These pathogens included bovine leukemia virus (BLV), bovine viral diarrhea (BVD), and *Mycobacterium avium* subsp. *paratuberculosis* (MAP), which were identified by serology using an enzyme-linked immunosorbent assay (ELISA) that detects antibodies specific to these pathogens as described [24].

### 2.3. Amplicon Library Processing

DNA was extracted from 660 samples recovered from cattle at each of the five farms using the QIAamp DNA Stool Mini Kit (QIAGEN; Valencia, CA, USA). DNA was extracted from 250 mg of feces or from the RAJ swabs stored at −80 °C. A fragment of approximately 569 bp from the V3–V5 hypervariable region of the bacterial 16S rRNA gene was amplified using the linker primer 357F (5′-CCGTCAATTCMTTTRAGT-3′) and the reverse primer 926R (5′-CCTACGGGAGGCAGCAG-3′). A sample-specific barcode of 6–8 nucleotides was used to sequence samples in parallel on a single 454-sequencing plate. The amplification and pyrosequencing methods were described in our prior study [14].

The raw pyrosequencing reads were analyzed using the Quantitative Insights Into Microbial Ecology (QIIME) software v.1.9.1 workflow for 454 data [26]. First, the sequences were demultiplexed based on the nucleotide barcode and quality filtered using ‘split_libraries.py’. Then, *de novo* operational taxonomic units (OTUs) were identified with ‘pick_de_novo_otus.py’, and the taxonomy was assigned with the SILVA database (v132) [27]; OTUs were clustered at 97% similarity. All samples were denoised with ‘denoise_wrapper.py’ to reduce the amount of erroneous OTUs [28], and OTU chimera detection and filtering was done with VSEARCH [29]. Lastly, the sequences were aligned with ‘align_seqs.py’ [30] and were converted into a phylogenetic tree using the QIIME 2 plugin ‘qiime fragment-insertion sepp’ [31,32,33].

### 2.4. Microbiome Analyses

The OTU table, taxonomy, metadata, and phylogenetic tree were imported into the R package Phyloseq v.1.24.2 [34]. Mitochondria and chloroplast OTUs were removed. Library rarefaction was applied to calculate alpha and beta diversities among samples. Alpha diversity was estimated to determine the richness and evenness of OTUs with the Shannon index, and richness based on the presence of rare OTUs (singletons and doubletons) was estimated with Chao1. The Wilcoxon and Kruskal–Wallis non-parametric tests were used to compare the alpha diversity estimates among LSP and HSP farms and STEC shedders and non-shedders. Beta diversity was also analyzed to compare the microbiome composition among groups using Bray–Curtis dissimilarity and weighted UniFrac distances [35]. The ordination was calculated by principal coordinate analysis (PCoA), which was plotted with two axes. The difference between categorical variables and the microbial profiles were calculated with permutational multivariate analysis of variance (PERMANOVA) with 999 permutations using the Vegan package v.2.5–6 [36].

The differentially abundant taxa analysis was performed using differential expression analysis based on the negative binomial distribution DESeq2 (v.1.30.1) with default settings [37]. The R package metacoder v.0.3.3 [38] was used to visualize the taxa abundance as “heat trees” with the proportion of bacterial families. Phylogenetic Investigation of Communities by Reconstruction of Unobserved States (PICRUST2) [39] was also used to predict metabolic pathways and enzymes based on 16S rRNA gene sequences. Linear discriminant analysis (LDA) effect size (LEfSe) v.1.0 [40] was used to identify differentially abundant pathways.

## 3. Results

### 3.1. Farm Characteristics

Three dairy and two beef herds were included in this study based on their STEC prevalence [11]. One beef and two dairy farms (1B, 2D, and 4D) were classified as LSP farms, which were compared with HSP farms consisting of one beef and one dairy farm (8B and 9D). The five farms represented varying breeds and sizes with different healthcare and management practices (Table 1). Notably, the LSP farms fed animals a lower percentage of forage in their diet (15–65%) and used anthelmintics, while HSP farms used a diet almost exclusively based on forage (80–100%) and did not provide anthelmintic treatments. Specific characteristics unique to the dairy farms, such as number of milks per day, days in milk (DIM), and number of lactations are shown in Table A1.

### 3.2. Sequencing Results

Twenty-eight 454-sequencing plates containing 660 samples yielded 1,937,794 reads of 569 bp paired-end fragments of the 16S rRNA gene. After trimming and quality filtering the sequences, the library size varied from 650 to 16,786 with a median library size of 2332 sequences per sample. Following the *de novo* clustering, denoising, filtering chimeras, and removal of chloroplast and mitochondria OTUs, 15,158 OTUs were detected.

### 3.3. Hindgut Microbiota Composition

**Figure 1:**
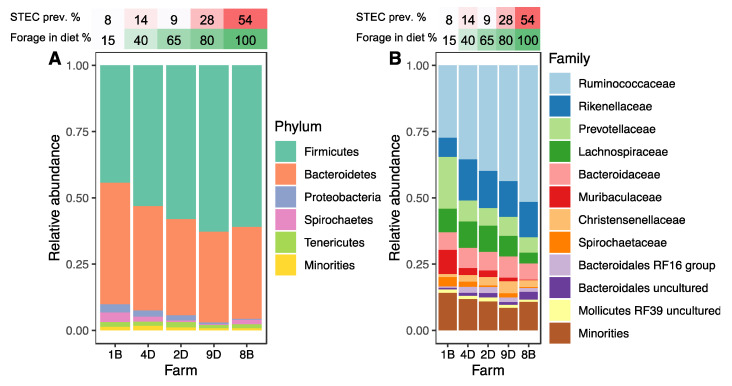
Hindgut microbiota composition of cattle from five farms with varying percentages of forage in the diet. Stacked bar charts show the relative abundance of bacterial (**A**) phyla and (**B**) families per farm. Less abundant taxa were grouped together and named “Minorities”.

The microbiota profiles for the 256 paired fecal grab (FG) and rectal-anal junction (RAJ) samples were similar (*p* > 0.05), and hence, these samples were combined into a single group representing the hindgut for downstream analyses (Figures S1 and S2). Overall, the hindgut microbiota was dominated by two phyla, Firmicutes (54.6%) and Bacteroidetes (38.9%), although varying proportions of other phyla were detected across farms with different forage percentages (Figure 1A). Indeed, the percentage of forage in the diet significantly influenced the microbial composition. Farms with low forage diets, for instance, had a lower abundance of Firmicutes and a higher abundance of Bacteroidetes (*p* < 0.0001). Classifying by family identified similar differences across farms with Ruminococcaceae predominating but increasing with the forage percentage (*p* < 0.001) (Figure 1B). Several additional bacterial families were significantly correlated with the percentage of forage in the diet (Table S2).

### 3.4. HSP Farms Characterized by Forage-Dominant Diets Exhibited Higher Alpha Diversity and a Distinct Microbiota Structure

**Figure 2:**
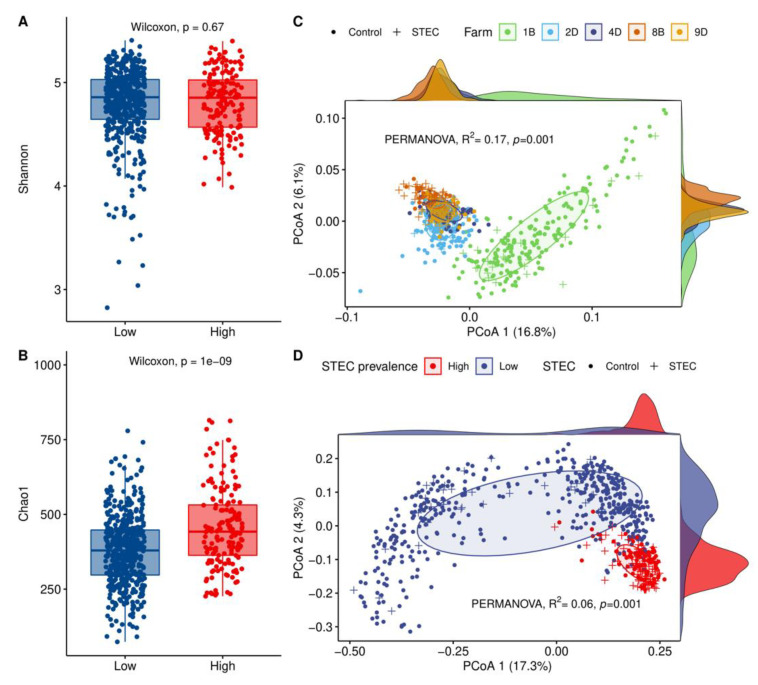
Cattle-hindgut microbiota alpha and beta diversities among farms with a low STEC prevalence (LSP) and high STEC prevalence (HSP). LSP farms (1B, 2D, and 4D) were combined as were the two HSP farms (8B and 9D) to evaluate the alpha diversity using (**A**) the Shannon index and (**B**) the Chao1 index. Beta diversity was evaluated by performing a (**C**) principal coordinate analysis (PCoA) of weighted UniFrac distances and/or a (**D**) PCoA of Bray–Curtis dissimilarity. The former plotted each farm separately along with the STEC shedders (+) and non-shedders (control, black circle), while the latter compared farms with LSP versus HSP.

To assess the association between STEC prevalence and the hindgut microbiota among farms, we analyzed the Shannon and Chao1 indices for alpha diversity and the Bray–Curtis dissimilarity and weighted UniFrac for beta diversity. HSP farms exhibited greater richness than LSP farms, although no significant difference was observed using the Shannon index (*p* = 0.67) (Figure 2A). The Chao1 index, however, detected significantly greater diversity in HSP farms (*p* = 1 × 10^−9^), indicating that a high number of OTUs were present in low proportions (singletons and doubletons) in the two HSP farms (Figure 2B). Notably, when comparing the alpha diversity indices between herds, the lowest and the highest OTU richness corresponded to farms 1B and 8B, respectively, which also had the lowest and the highest STEC prevalence (Figure S3). When the farms were plotted separately to evaluate beta diversity, the PCoA plot of weighted UniFrac distances showed that the microbial communities from LSP farm 1B were the most divergent relative to the other four farms (Figure 2C). Comparatively, the farms were classified by STEC prevalence, and a Bray–Curtis dissimilarity PCoA was generated (Figure 2D). This plot shows that cattle from the two HSP farms had a more similar microbiota structure that was significantly different than the microbiota profiles observed in the three LSP farms (PERMANOVA, *p* < 0.0001). HSP microbiota clustering was strongly associated with dominant forage diets (Figure S4).

### 3.5. Hindgut Microbiota Diversity Comparisons between STEC-Positive Samples and Controls

**Figure 3:**
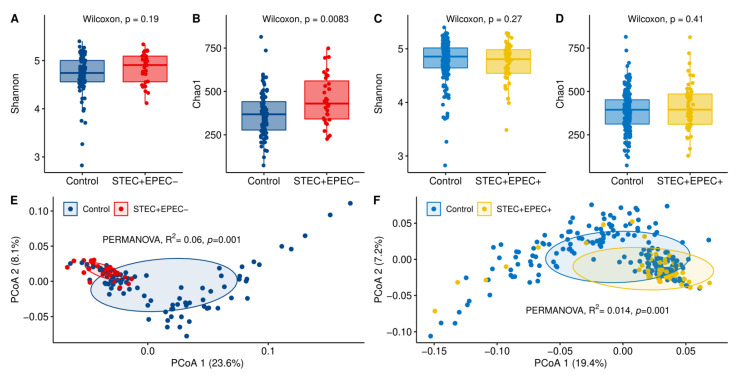
Cattle-hindgut microbiota diversity comparisons between animals shedding STEC or EHEC and non-shedders (controls). The alpha diversity was evaluated for 31 STEC-positive (*stx*-positive, *eae*-negative) cattle (red dots) for comparison to 85 STEC/EHEC-negative control cattle (blue dots) using the (**A**) Shannon and (**B**) Chao1 indices, while (**E**) beta diversity was examined using a principal coordinate analysis (PCoA) of weighted UniFrac distances. The (**C**) Shannon and (**D**) Chao1 alpha diversity indices as well as a (**F**) PCoA for beta diversity were also evaluated for the 52 EHEC shedders (*stx*-positive, *eae*-positive; light blue dots) for comparison to a larger sample of 205 randomly selected non-shedders (yellow dots) from the five herds.

The microbiota profiles of STEC-shedders and non-shedders were examined in more detail regardless of farm. To do this, the animals were split into two comparison groups based on the recovery of isolates positive for *stx* and/or *eae* for comparison to cattle with isolates that were negative for both virulence factors; 3–4 control animals were randomly selected from each of five farms for each case included in the analysis. The first comparison group examined 31 STEC-positive (*stx* only) cattle from HSP farms and 85 STEC/EHEC-negative (control) cattle including 20 from HSP and 65 from LSP farms. For the second comparison group, EHEC-positive (*stx* and *eae* only) cattle (*n* = 52) were compared to a larger set of STEC/EHEC-negative control animals (*n* = 205). EHEC comparison included cattle from HSP farms (cases = 34, controls = 20) and LSP herds (cases = 18, controls = 185). These groups excluded animals positive for BLV and MAP because they had significantly different microbiota profiles based on the alpha and beta diversity metrics (*p* < 0.05) (data not shown). Indeed, the exclusion of samples from MAP- and BLV-positive cattle was necessary given that prior studies showed that these pathogens were associated with important gut microbiome changes [41,42]. Notably, the STEC shedders possessing *stx* only had higher microbiota richness than the non-shedders (Shannon, *p* = 0.19; Chao1, *p* = 0.008), though only the Chao1 metric was significant (Figure 3A,B). By contrast, no difference in alpha diversity was observed when the EHEC shedders (*stx*-positive and *eae*-positive) were compared to the STEC/EHEC-negative controls (Shannon, *p* = 0.27; Chao1, *p* = 0.41) (Figure 3C,D). The microbiota structure of the STEC and EHEC shedders, however, was significantly different from that of the controls in both comparison groups (PERMANOVA, *p* < 0.001) (Figure 3E,F).

### 3.6. STEC Carriers from Farms with LSP but Not HSP Showed Changes in Microbiota Diversity over Time

**Figure 4:**
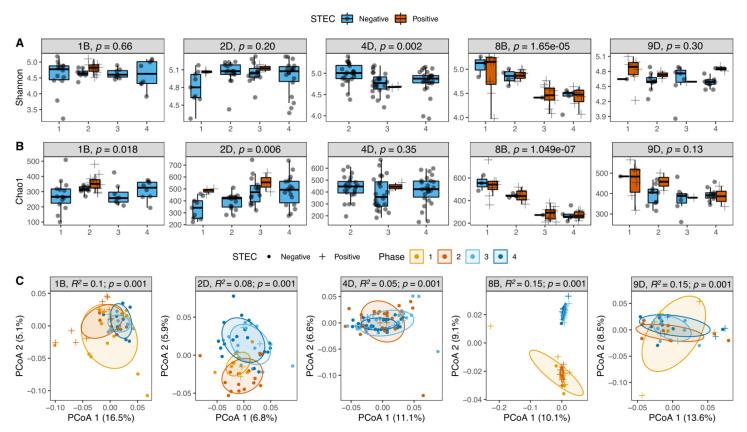
Temporal dynamics in microbiota diversity among 59 cattle from five farms. Alpha diversity was compared using the (**A**) Shannon and (**B**) Chao1 indices by farm and sampling period. Each box represents a different farm with the numbers on the x-axis representing the four sampling visits; significant differences were detected using the Kruskal–Wallis test. (**C**) Beta diversity was also examined using a principal coordinate analysis (PCoA) of the weighted UniFrac distances; PERMANOVA results (*R*^2^ and *p*-value) were calculated. STEC shedders (+) and non-shedders (circles) were plotted by phase, which is represented by four different colors.

Among the 59 cattle evaluated longitudinally from five farms (Table S1), we sought to determine how STEC shedding impacted the microbiota composition in the hindgut. Despite finding no significant modifications due to diet, management, and environmental conditions (Table S3) as reported within the farms over time, all cattle had significant differences in the alpha and beta diversities across the four samplings or phases. In general, the STEC-positive samples exhibited higher alpha diversity within each phase, particularly among the three LSP farms (Figure 4A,B); however, none of the animals from the LSP farms carried STEC in more than one phase. By contrast, the cohorts from the HSP farms, 8B and 9D, had a high proportion of cattle shedding STEC in all four phases. Each animal from farm 8B (*n* = 13) shed STEC in two or more phases. Indeed, the biggest difference in alpha diversity over time was observed in the HSP 8B farm as both the Shannon and Chao1 indices were significantly different between STEC shedders and non-shedders over time. In farm 9D, 25% of animals shed the pathogen in two phases, while the remaining cattle shed in just one phase, and the alpha diversity was steady.

Differences in the microbiota composition or beta diversity, as determined by the PCoA, were also observed across samplings at each of the five farms evaluated (Figure 4C). Curiously, the Angus farm (8B) had two microbiota profiles that were not associated with STEC shedding status. In farm 8B, the microbiota profiles in phases 1 and 2 were similar, highly diverse, and dominated by Bacteroidetes (*log2 fold change* = 0.22; *p* = 0.0009). The microbiota profiles in phases 3 and 4, however, were distinct from those observed in phases 1 and 2, which coincided with a decreased alpha diversity and an increased abundance of Firmicutes (*log2 fold change* = 0.15; *p* = 0.05). Despite these differences, the proportion of STEC-positive animals in farm 8B was steady across the four phases.

### 3.7. Differentially Abundant Taxa among the STEC Shedders from Dairy Farms

**Figure 5:**
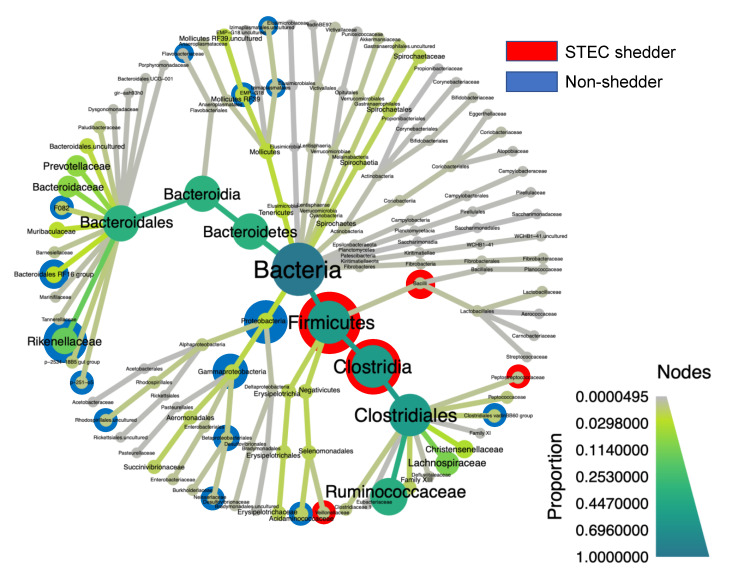
Heat tree showing the differentially abundant taxa found in the hindgut microbiota among STEC shedders (red) and non-shedders (blue) at three dairy farms. Only those taxa with a *p*-value lower than 0.01 were included. Node size and color correspond to the relative abundance at each taxonomic level.

Among the three dairy farms, STEC shedders had a significantly greater abundance of Firmicutes and a lower abundance of Proteobacteria than the non-shedders (*p* < 0.01) (Figure 5). No taxa were identified when comparing between STEC-shedders and non-shedders among the dairy farms when controlling for farm. A total of 30 genera were found to be differentially abundant between STEC carriers and non-carriers (Table S4).

### 3.8. Taxa Correlated with Factors Associated with STEC Carriage

**Figure 6:**
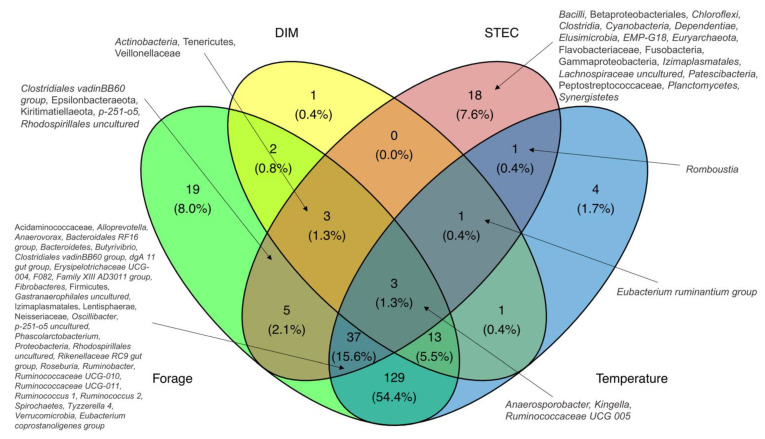
Venn diagram showing the number of differentially abundant genera associated with STEC shedding (STEC), percentage of forage in diet and risk factors of STEC in dairy farms (temperature and days in milk). Percentages represent the proportion of taxa intersected between different variables.

Next, we analyzed how the microbiota composition is impacted by previously identified risk factors of STEC shedding in cattle [11] including maximum temperature 5 days prior to sampling, days in milk (DIM), and the number of lactations (Figure 6, Table S5). Notably, temperature increases were associated with a differential abundance of 189 taxa including 42 observed among the STEC shedders (Table S4). Similarly, the number of DIM was significantly correlated with 24 differentially abundant genera including those associated with STEC carriage such as *Actinobacteria*, *Anaeroporobacter*, *Kingella*, *Ruminococcaceae UCG-005*, Tenericutes, Veillonellaceae, and the *Eubacterium ruminantium* group. Finally, seven taxa were correlated with the number of lactations, including an increase of *Kingella* and Neisseriaceae and decrease of Lentisphaerae and *Ruminococcaceae UCG-011* as observed in non-STEC shedders. Forage percentage in diet across farms was associated with changes in 211 taxa of which 48 were associated with STEC shedding (Figure 6).

## 4. Discussion

Preventing STEC shedding in livestock could significantly reduce the number of human infections. In this study, we sought to determine differences in the gut microbiota of bovines from farms with a low versus high STEC prevalence. In addition, we explored factors that could affect the microbial composition and contribute to STEC shedding. The diversity and composition of 16S *rRNA* sequences of 660 hindgut samples from five cattle farms (beef and dairy) were analyzed in this study. Cattle from HSP farms, characterized by being fed a high percentage of forage in their diet (80–100%), had a significantly higher richness of OTUs than LSP farms, which had a lower proportion of forage in the diet (15–65%). Longitudinal analysis showed that most STEC shedders from LSP farms had a greater microbial diversity than non-shedders; however, cattle from HSP farms showed changes in the microbial diversity that were not linked to the STEC carriage. Furthermore, bacterial taxa associated with STEC shedding were also correlated with diet and previously described risk factors of STEC. Meanwhile, significant differences in predicted metabolic pathways in animals from LSP and HSP farms reflect functional differences of the microbiota between herds that could affect STEC colonization.

The overall bacterial composition of the hindgut microbiota was similar to that in prior studies where Firmicutes and Bacteroidetes were the dominant taxa [43,44]. Though farm-specific composition was identified as previously observed in ten dairy farms with different housing, diet, and husbandry [43]. Notably, unlike LSP farms, HSP farms had in common a high-forage diet and did not administer anthelmintic treatment, which could affect the microbiome composition. The effect of diet in the gut microbiota was previously studied in cattle, where different ratios of forage:concentrate impacted changes in the microbiota [45,46,47]. As previously observed, a grain-based diet was associated with a higher abundance of Proteobacteria and lower abundance of Bacteroidetes [45,47]. Meanwhile, forage-dominant diets were associated with a higher abundance of Firmicutes, Ruminococcaceae and *Paludibacter*, which have a critical function degrading forage [45,47]. Dietary interventions in beef cattle have been suggested to reduce the prevalence of STEC O157 as a preharvest intervention [48,49]. However, without knowledge of the microbiome and ecological interactions, those studies had conflicting results [48]. Furthermore, the effect of helminths in the cattle’s microbiome has not yet been studied. Anthelmintic treatment in dogs and horses, for instance, was not associated with shifts in the microbiome composition [50,51]. Humans treated with albendazole, however, had a higher abundance of Clostridiales and a lower abundance of Enterobacteriales [52]. Meanwhile, helminthic infections were associated with a lower abundance of Lachnospiraceae in the human gut microbiome [53].

Higher alpha diversity identified among some STEC shedders has been observed in previous studies in both beef [16,54] and dairy cattle [22]. By contrast, some reports have found that STEC carriage in individual cattle was associated with lower alpha diversity [17,20,23,55]. Two of these reports were carried out in beef herds, where correlations between bacterial richness, STEC enumeration, and age (weaning to one year) were compared. While the authors found that older animals had higher microbial diversity and younger animals (1–6 months) shed a higher number of STEC, the correlation between microbiome diversity and STEC shedding reflects factors related to age and dietary changes [17,20]. Other reports in dairy cattle that identified a lower alpha diversity in the gut microbiome were focused on shedding of STEC O157, the serogroup associated with a higher number of hospitalizations in humans. Stenkamp-Strahm et al. (2017) detected a weak association after removing outliers [23], while Mir et al. (2020) identified lower alpha diversity in STEC carriers only after vaccination and oral challenge with O157. Hence, these findings indicate that O157 carriage did not directly affect the microbiota but that vaccination for O157 can alter the microbiota diversity [55]. Indeed, the longitudinal analysis of HSP farms examined herein and in our prior study [56], showed similar microbiota shifts as those observed in O157 vaccinated cattle, suggesting that STEC re-infection in cattle can be followed by a lower alpha diversity.

The microbiota composition of STEC-positive samples mostly overlapped with that of negative samples in the PCoA. Nevertheless, differentially abundant taxa have been documented among STEC shedders and non-shedders [17,19,23,24,54]. Zhao et al. (2013), for instance, found that butyrate-producing species were more abundant in low-STEC-shedding cattle and were critical in avoiding RAJ lesions [17], suggesting the role of certain taxa as “inhibitors” or “promoters” [17]. Contrasting results have been observed among studies, but in general, there is a consensus that STEC shedders have a higher proportion of members from the order Clostridiales, the dominant order found in the bovine gut microbiome [18,21,22,23,24,54,55,57]. Consistent with other reports, a lower abundance of Proteobacteria was observed in STEC shedders [22,55]. Varying results in differentially abundant taxa among STEC shedders and non-shedders denote a high variability between species and strains within taxa, as well as differences between study approaches and farms.

In this study, the main microbial biomarkers of STEC shedders were *Romboutsia* and *Alloprevotella,* implicated in the production of C_12_–C_19_ fatty acids [58] and succinic acid [59], respectively. Other genera significantly higher in STEC shedders were associated with sugar fermentation and the production of acetic, formic, propionic, and succinic acids [60,61,62]. By contrast, the main biomarkers of non-shedders were *Kingella*, *Bacteroidales p-251-o5* and *Anaerosporobacter*. In humans, *Kingella* is implicated in invasive infections due to its cytotoxicity [63]. Butyrate-producing bacteria, including *Butyrivibrio*, *Oscillibacter*, *Roseburia,* and *Ruminobacter*, were also found to be associated with non-shedders [64,65]. These families have previously been linked to a healthy human gut microbiota and were suggested to play a role in preventing chronic intestinal inflammation [66]. The functional role of these taxa in the bovine microbiome or in immunomodulation as well as the correlation with pathogen colonization, however, requires further investigation.

Differences in predicted metabolic pathways observed between LSP and HSP farms suggest that distinct functional microbiomes could favor STEC carriage. For instance, metabolic-pathway prediction showed important differences between HSP and LSP farms associated with diet, where HSP farms had higher oxidation, production of short-chain fatty acids, degradation, and fermentation than LSP farms, which had higher biosynthesis of amino acids and sugar degradation (Figures S5 and S6). Enhanced fermentation and fatty acid production in HSP farms could be influenced by forage-dominant diets. Comparatively, the LSP farms had higher amino acid biosynthesis, suggesting differing amino acid availability in diets within the LSP and HSP farms. Higher inositol degradation in LSP farms shows enhanced cleavage of phospholipid membranes that generate cell signaling molecules (i.e., inositol phosphate and diacylglycerol) important for microbe–host interactions [67]. Indeed, distinct metabolic profiles were suggested to be influenced by the diet, as a higher grain diet lowered the ruminal pH and altered the abundance of several metabolites including short-chain fatty acids, amino acids, ethanol, endotoxins, and biogenic amines [68]. Increasing amounts of grain in diets are also correlated with increasing concentrations of ethanolamine [68], the main product of enterocyte membranes, which is degraded to ethanol and acetate. Studies have shown that both *Salmonella* spp. and STEC O157 can use ethanolamine as a nitrogen source to outcompete commensal bacteria [69,70]. These studies, however, were carried out in vitro under aerobic conditions, unlike the intestinal environment.

Microbiota diversity and STEC shedding are dynamic over time as different patterns were observed between farms with a low and high STEC prevalence. Longitudinal studies in cattle found that the stability of the gut microbiota diversity and composition depends on the diet [71]. Once the animals are adapted to a specific diet, the microbial communities are relatively stable [71]. Unlike farms with low STEC prevalence, animals from farms with high STEC prevalence had access to pasture and a diet primarily based on forage. The grazing behavior and differences in forage composition in farms with high STEC prevalence could explain the high variability over time in their microbiota and a higher STEC detection. We identified that cattle from farms with low STEC prevalence only shed the pathogen once in an 8–12-week period. Meanwhile, most of the cattle from farms with a HSP prevalence shed the bacteria more than once. We also identified STEC super shedders only in the HSP farms (data not shown). A longitudinal study carried in dairy cattle for a 12-month period identified a very low number of STEC super-shedders in farms with a low STEC prevalence (3.5–5%), and those animals only shed the bacteria once a year [72]. Other studies have reported that the within-farm proportion of super-shedders ranges from 3.8% to 25%, highlighting the importance of farm-specific differences on STEC prevalence and shedding levels [73,74,75].

The bioinformatics pipeline used in this study was designed to improve the quality of the pyrosequencing results. We used SATé-Enabled Phylogenetic Placement (SEPP) trees to more accurately identify the phylogenetic relationships between OTUs by including sequences of known species [33]. SEPP trees are strongly recommended to avoid incorrect results driven by erroneous phylogenetic placements as observed in de novo trees [31]. Using SEPP trees was critical to account for differences in the beta diversity using weighted UniFrac metrics and to predict metabolic pathways with PICRUSt2. In addition, we used non-linear approaches to identify differentially abundant taxa. Linear discriminant analyses, which assume normality, showed similar results to those of DESeq though fewer taxa were identified as significantly different.

Prior studies looking for associations between the bacterial composition and STEC shedding used different techniques to identify taxa including denaturing gradient gel electrophoresis (DGGE) [17], pyrosequencing [16,18,20,24,54], and Illumina dye sequencing [19,21,22,23,55,57]. Despite differences in the pipeline, commonalities in the microbiota composition and differentially abundant taxa present in STEC shedders were observed across studies. As the sequencing techniques and bioinformatic tools evolve rapidly, high-resolution results will help to better understand more complex relationships within the microbiome. Along with defining the taxonomic composition, it is important to characterize molecular interactions between microorganisms and hosts by identifying KEGG pathways and metabolites that are common among STEC-positive cattle.

Nonetheless, this study has several limitations, and hence, the data should be interpreted carefully. For instance, we compared animals from cattle farms with different genetic backgrounds, diet, housing, locations, and husbandry practices, which could be confounding factors that also influence microbiota diversity and composition between farms. Furthermore, using pyrosequencing, we were able to detect differences in numerically dominant taxa, limiting the identification of low-abundant taxa that could also play a key role in defining the composition of the microbiota. For instance, the proportion of *Escherichia* was very low and absent in a large proportion of samples through pyrosequencing analysis. Metabolic pathways predicted from 16S rRNA gene sequencing showed significant differences between HSP and LSP farms (Figure S5) and between STEC shedders and non-shedders (Figure S6). These predictions, however, are not entirely accurate as they are based on metabolic reconstruction of a few representative species and do not account for genome differences between closely related strains. Despite this limitation, they provide clues that can be used to guide future studies aimed at defining the function of the cattle gut microbiome in the presence and absence of STEC. Future studies should also use a longitudinal approach and consider the within-farm STEC prevalence to better identify changes in the microbiome among shedders. Understanding the role that anthelmintics play in STEC shedding should also be addressed, while metagenomic and metabolomic data should be evaluated to identify key metabolites, genes, and bacterial species that could inhibit STEC colonization and boost the gut immune response.

## 5. Conclusions

This study suggests that STEC carriage in cattle is favored by highly diverse microbiota profiles, which are associated with forage-dominant diets. In addition, multiple factors affect the abundance of taxa associated with STEC shedding in dairy farms, including diet, number or lactations, DIM, and warm temperatures. Identifying healthy microbiomes could guide novel husbandry decisions that aim to decrease levels of pathogen shedding.

## Figures and Tables

**Table 1:** Characteristics of each cattle farm examined.

Feature	Farm				
	2D	4D	9D	1B	8B
Breed	Holstein	Holstein	Holstein	Crossbreed	Angus
Herd	Dairy	Dairy	Dairy	Beef	Beef
Herd size	320	3000	243	136	54
STEC prevalence (%)	8.7	13.8	28.0	8.2	53.7
STEC prevalence classification	LSP	LSP	HSP	LSP	HSP
No. of samples	213	81	77	206	83
Fecal grab	48	40	77	60	83
Recto-anal junction	165	41	0	146	0
Mean age days (SD ^a^)	1382 (476)	NR ^b^	1362 (522)	372 (19)	442 (17)
Housing	Free stall; tie stall	Free stall	Access to pasture/dry lot; free stall	Feedlot	Loose house
Diet % (SD)					
Forage	65.01 (18.76)	40.62 (9.47)	80 (0)	15 (0)	100 (0)
Concentrate	34.99 (18.76)	59.38 (9.47)	20 (0)	85 (0)	0
Corn silage	29.06 (8.82)	41.7 (3.74)	0	15 (0)	0
Cotton seed	1.60 (2.60)	0	0	0	0
Rumensin	No	Yes	No	Yes	No
Roughage, protein	No	No	Yes	No	No
Season ^c^	Summer	Summer	Summer	Spring	Summer
Temperature (°C)	25	25	36	4.4	36
Humidity (g/m^3^)	66	68	31	75	42
Temp. max. 5 days ^d^	23.44	29.89	37.11	20.33	29.33
Temp. avg. 5 days ^d^	19.22	16.89	30.11	13.89	22.78
Treatment					
Anthelmintic	Yes	Yes	No	Yes	No
Respiratory	Ceftiofur, florfenicol	Ceftiofur	None	Ceftiofur, tulathromycin	Florfenicol
Foot infection	Copper sulfate, penicillin	Copper sulfate	Copper sulfate, oxytetracycline, ceftiofur	Oxytetracycline	Ceftiofur

^a^ SD = standard deviation; ^b^ NR = not reported; ^c^ during sample collection; ^d^ temperature five days prior to sample collection.

## Data Availability

The data presented in this study are openly available in GitHub at https://github.com/karla-vasco/cattle.microbiome-STEC (published on 23 June 2021).

## Supplementary Materials

The following are available online at https://www.mdpi.com/article/10.3390/microorganisms9081737/s1, Figure S1: Paired comparison of fecal grab (FG) and recto-anal junction (RAJ) microbiome alpha and beta diversities. Figure S2: Paired comparison of fecal grab (FG) and recto-anal junction (RAJ) microbiome alpha and beta diversities per farm and sampling phase. Figure S3: Cattle-hindgut microbiota alpha diversity among five farms. Figure S4: Principal coordinate analysis showing differences in the microbiome composition associated with the percentage of forage in diet. Figure S5: Linear discriminant effect sizes of metabolic pathways inferred from 16S rRNA sequences between farms with low and high STEC prevalence. Figure S6: Linear discriminant effect sizes of metabolic pathways inferred from 16S rRNA sequences between STEC shedders and non-shedders. Table S1: Number of animals sampled longitudinally per farm and number of days from each phase. Table S2: Bacterial families differentially abundant in the bovine-gut microbiota of animals fed with different percentages of forage in diet. Table S3: Temperature (°C) and relative humidity (RH) in the cattle farms during four sampling phases. Table S4: Differentially abundant taxa identified between STEC shedders and non-shedders among dairy farms. Table S5: Taxa correlated with factors associated with STEC carriage.

## Appendix A

**Table A1:** Characteristics of the three dairy farms examined in this study.

Feature	Dairy Farm		
	2D	4D	9D
No. of milkings/day	2 times	3 times	2–3 times
DIM (SD)	206.7 (134)	259 (118.5)	195.25 (136.43)
No. of lactations			
0 (No. of cows)	4	0	0
1 (No. of cows)	80	36	34
≥2 (No. of cows)	141	86	43
Dry (No. of cows)	5	9	0
Treatment			
Clinical mastitis	Ceftiofur, pirlimycin hydrochloride, penicillin, ampicillin, oxytetracycline, sulfadimethoxine	Penicillin G procaine, ceftiofur, pirlimycin hydrochloride, amoxicillin	Ceftiofur
Metritis	Oxytetracycline, penicillin	Ceftiofur	Ceftiofur, isoflupredone acetate
Dry	Penicillin-novobiocin, Penicillin-dihydrostreptomycin	Penicillin-dihydrostreptomycin, Orbeseal	None

No. = number; DIM = days in milk; SD = standard deviation.
