# Supplementary material for: Characterizing the Cattle Gut Microbiome in Farms with a High and Low Prevalence of Shiga Toxin Producing Escherichia coli
Source: Microorganisms. 2021 Aug 14;9(8):1737. doi: 10.3390/microorganisms9081737 (PMC8399351; doi:10.3390/microorganisms9081737)
Supplement: Supplementary file 1 [file microorganisms-09-01737-s001.zip › microorganisms-1285616-supplementary.pdf]

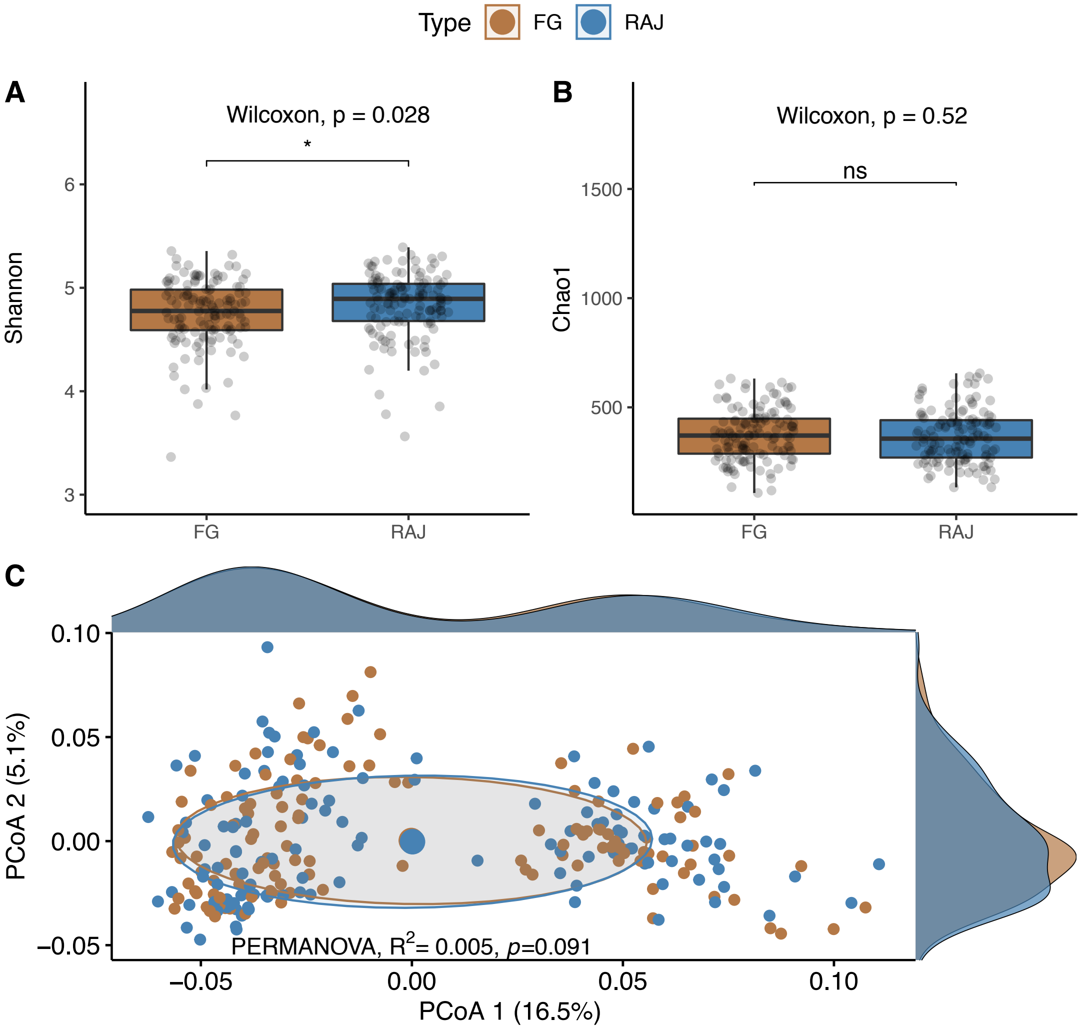

Figure S1. Paired comparison of fecal grab (FG) and recto-anal junction (RAJ) microbiome alpha and beta diversities. A) Shannon index boxplots. B) Chao1 index boxplots. C) Principal coordinate analysis of Weighted Unifrac ordination. The centroids of FG and RAJ distributions are shown with a bigger point size.

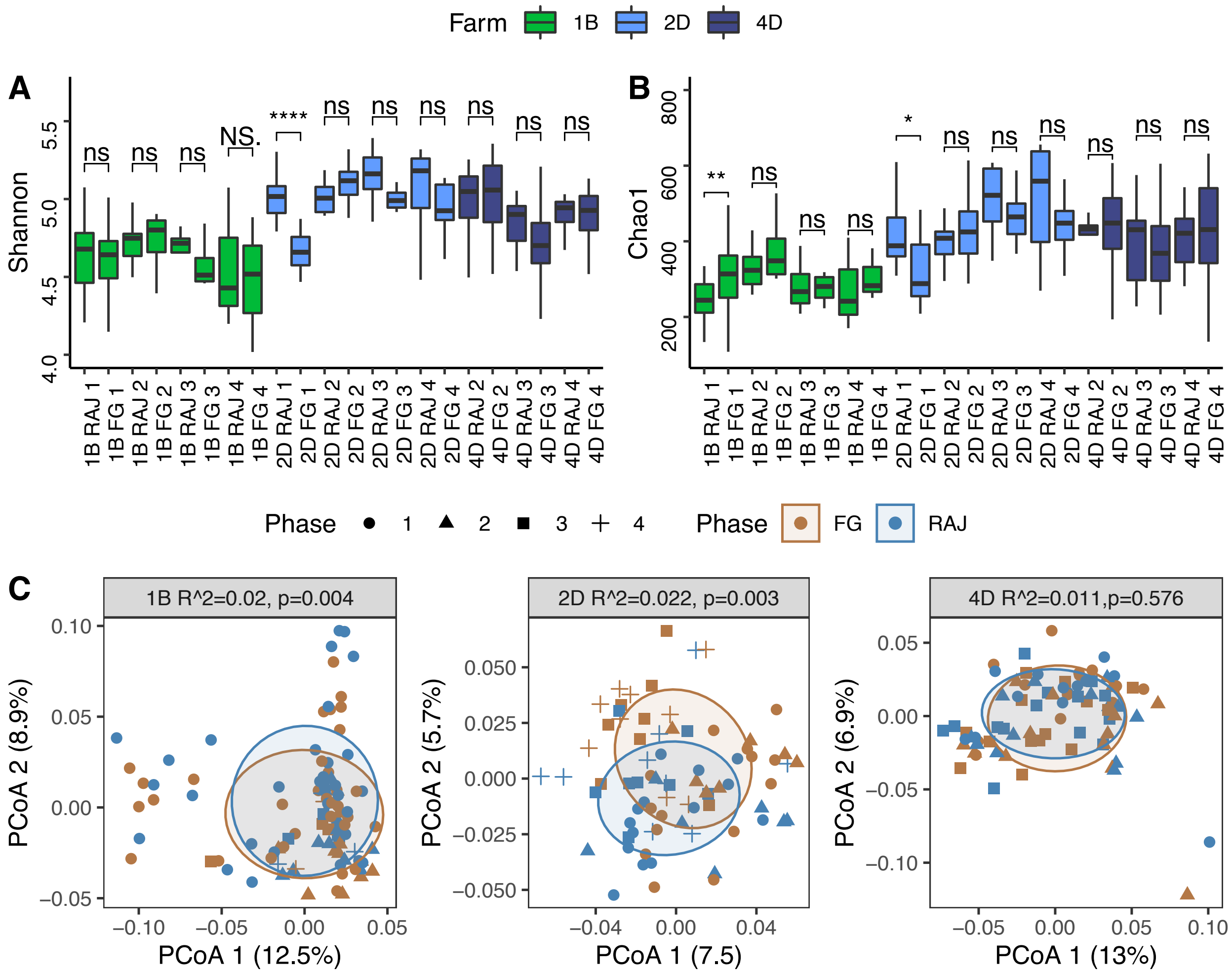

Figure S2. Paired comparison of fecal grab (FG) and recto-anal junction (RAJ) microbiome alpha and beta diversities per farm and sampling phase. The *p-values* in A and B were calculated with the Wilcoxon test and are represented with symbols "****" ≤ 0.0001, "*" ≤ 0.05, and "*ns*" not significant. A) Shannon index boxplots, B) Chao1 index boxplots. C) PCoA of Weighted Unifrac ordination; each box corresponds to a farm; the R^2 and *p-value* are from the PERMANOVA test between RAJ and FG.

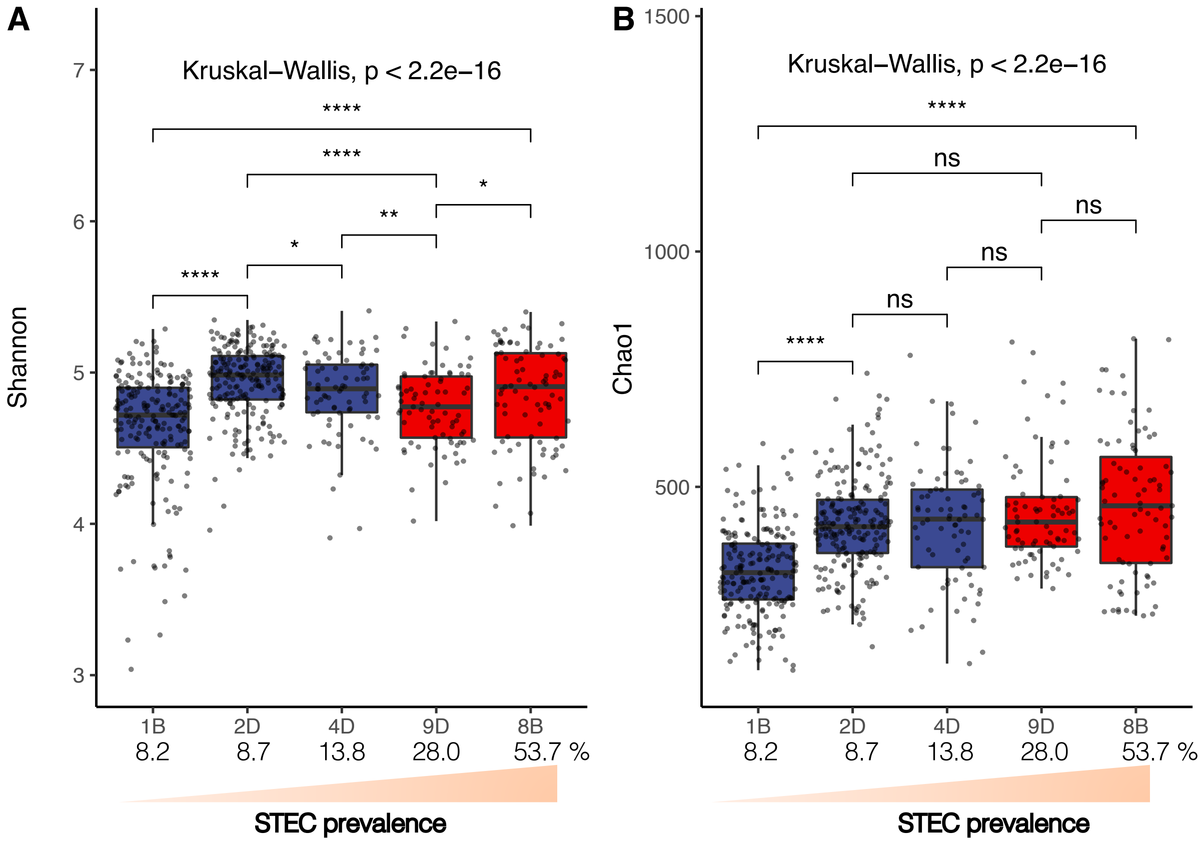

Figure S3. Cattle-hindgut microbiota alpha diversity among five farms. Farms classified as low STEC prevalence (LSP) are shown in blue, and farms with a high STEC prevalence (HSP) are in red. *p-value*: **** ≤ 0.0001, *** ≤ 0.001, ** ≤ 0.01, ns not significant.

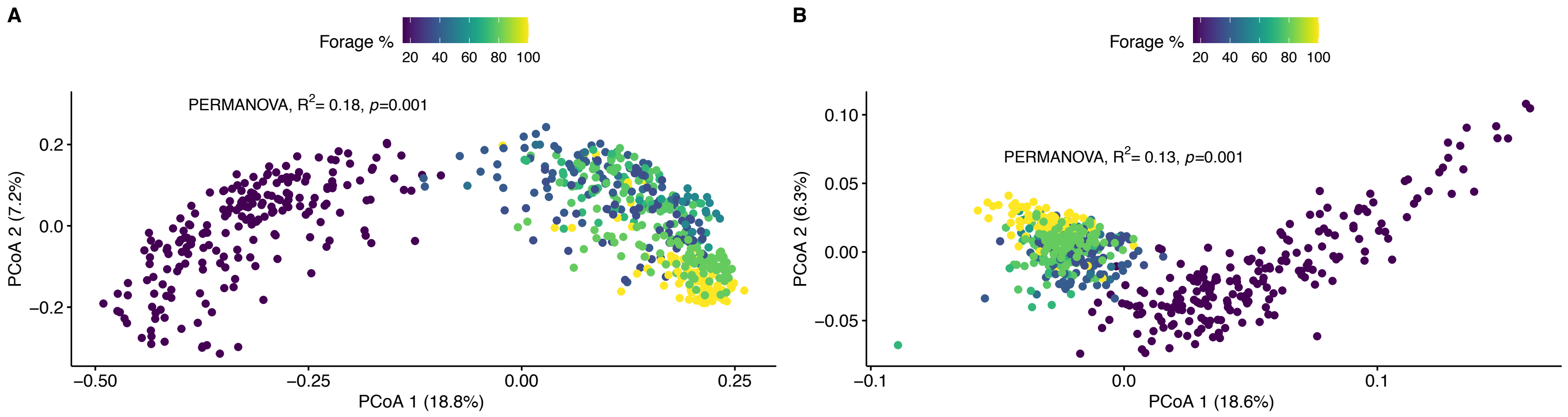

Figure S4. Principal coordinate analysis showing differences in the microbiome composition associated with the percentage of forage in diet. A) Bray-Curtis dissimilarity distances. B) Weighted Unifrac ordination.

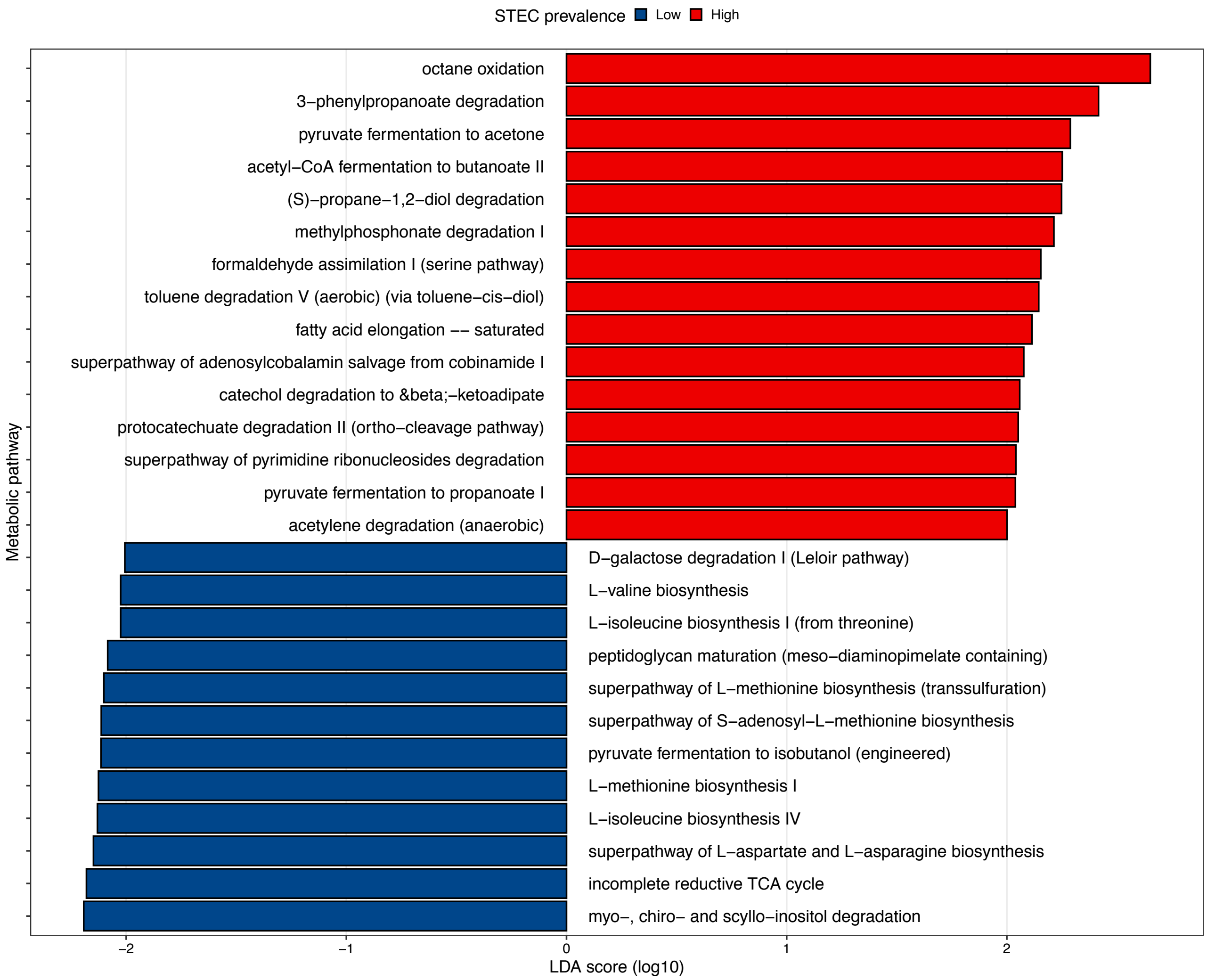

Figure S5. Linear discriminant effect sizes of metabolic pathways inferred from 16S *rRNA* sequences with PICRUSt2, between farms with low and high STEC prevalence. A total of 487 MetaCyc pathways were predicted from 16S *rRNA* sequences. LDA effect sizes of 5.5% of the pathways were significantly different between farms with a high and low STEC prevalence.

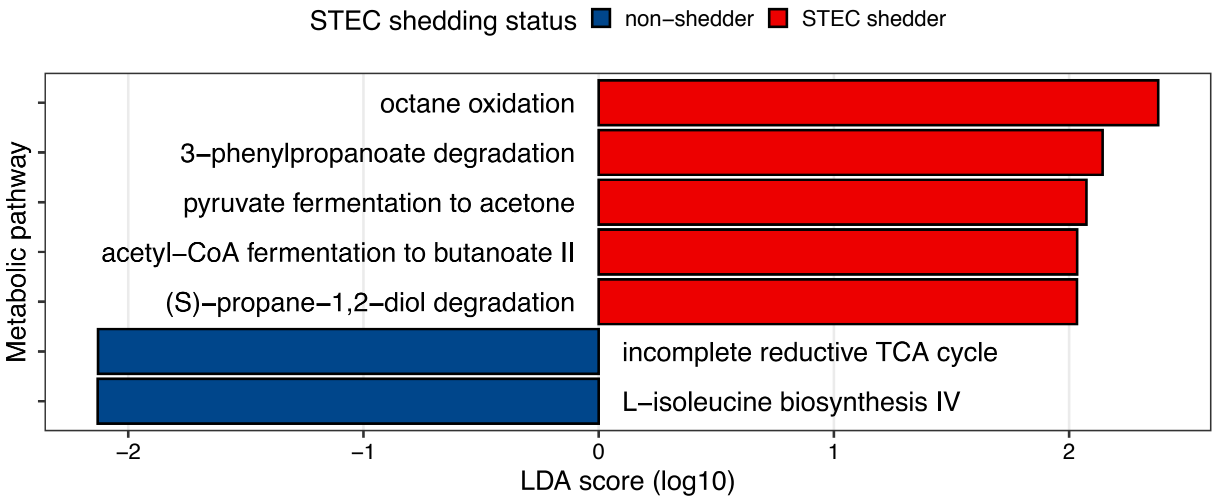

Figure S6. Linear discriminant effect sizes of metabolic pathways inferred from 16S *rRNA* sequences between STEC shedders and non-shedders.

Table S1. Number of animals sampled longitudinally per farm and number of days from each phase.

Farm	No. Animals	Days (No.)			
		Phase 1	Phase 2	Phase 3	Phase 4
1B	10	0	14	28	35
2D	10	0	10	38	67
4D	14	NA	0	21	42
8B	13	0	13	27	43
9D	12	0	29	43	49

Table S2. Bacterial families differentially abundant in the bovine-gut microbiota of animals fed with different percentages of forage in diet.

Family	Base Mean	log2 Fold Change	Lfc SE	Stat (Wald)	p-value	p-adj
Ruminococcaceae	925.388167	0.016471	0.0007213	22.8351089	2.05E-115	5.45E-114
Prevotellaceae	216.095712	-0.0143815	0.00087637	-16.410246	1.62E-60	2.14E-59
Rikenellaceae	280.427889	0.01722959	0.00105481	16.3343473	5.62E-60	6.62E-59
Lachnospiraceae	199.832725	-0.0032235	0.00061375	-5.2521776	1.50E-07	4.83E-07
Bacteroidaceae	158.256821	0.00520128	0.00117731	4.41791991	9.97E-06	2.78E-05
Muribaculaceae	82.2391578	-0.0407878	0.00118366	-34.459027	3.30E-260	3.50E-258
Christensenellaceae	58.2934835	0.02058984	0.0012825	16.0545191	5.31E-58	5.63E-57
Spirochaetaceae	38.7900993	-0.0285462	0.00210975	-13.53062	1.03E-41	7.81E-41
Acidaminococcaceae	21.0944346	-0.0117091	0.00101771	-11.505348	1.24E-30	6.92E-30
Succinivibrionaceae	19.2272586	-0.0390218	0.00304894	-12.798487	1.67E-37	1.18E-36
Planococcaceae	3.59067335	0.04576269	0.0056949	8.03572909	9.30E-16	3.52E-15
Clostridiaceae 1	10.3027843	-0.0332732	0.00220972	-15.057646	3.08E-51	2.72E-50
Veillonellaceae	5.7808527	-0.009967	0.00273246	-3.6476253	0.00026468	0.00066799
Bacteroidales RF16 group	39.2203283	0.02558717	0.00188224	13.5939779	4.35E-42	3.55E-41
Enterobacteriaceae	3.84993718	-0.0429341	0.00368522	-11.650361	2.28E-31	1.42E-30
Erysipelotrichaceae	25.0547186	-0.0057838	0.00099778	-5.7966039	6.77E-09	2.24E-08
p-2534-18B5 gut group	11.5089734	0.0655837	0.00367406	17.8504911	2.87E-71	5.06E-70
Gastranaerophilales uncultured	11.6396714	0.0086763	0.00191412	4.53278973	5.82E-06	1.67E-05
Fibrobacteraceae	2.37866879	-0.0196056	0.0045473	-4.3114863	1.62E-05	4.30E-05
Neisseriaceae	4.34797435	0.01931658	0.00612223	3.1551537	0.00160414	0.00377863
Tannerellaceae	11.9284829	-0.052279	0.0020561	-25.426363	1.29E-142	4.56E-141
Bacteroidales uncultured	37.365088	0.03093304	0.00202808	15.2523773	1.59E-52	1.53E-51
Family XIII	17.9457517	0.01915138	0.00110293	17.3641336	1.54E-67	2.34E-66
p-251-o5	6.50850208	0.00772406	0.00306325	2.52152497	0.01168474	0.0269257
F082	16.1470904	0.07207967	0.00266119	27.0855519	1.46E-161	7.72E-160
Anaeroplasmataceae	3.42937256	-0.0182558	0.00241217	-7.568193	3.78E-14	1.38E-13
Barnesiellaceae	3.84434886	0.01620163	0.00214236	7.56250378	3.95E-14	1.40E-13
Burkholderiaceae	3.97890005	-0.0218915	0.0019579	-11.181093	5.05E-29	2.67E-28
Clostridiales vadinBB60 group	14.6173565	0.00752756	0.00174331	4.31797553	1.57E-05	4.28E-05
Peptococcaceae	9.02004628	0.01093276	0.00126473	8.64431919	5.41E-18	2.29E-17
Rhodospirillales uncultured	7.76393104	0.01311455	0.00191066	6.86389013	6.70E-12	2.29E-11
Paludibacteraceae	5.83377217	0.04345508	0.00236365	18.3847304	1.74E-75	3.69E-74
EMP G18 uncultured	0.69694367	-0.0361119	0.00345028	-10.466371	1.23E-25	5.68E-25
Akkermansiaceae	2.83223975	0.02514747	0.0029462	8.53556341	1.39E-17	5.69E-17
Bifidobacteriaceae	0.5543199	-0.0232566	0.00603712	-3.8522622	0.00011703	0.00030257
Izimaplasmatales uncultured	4.60428363	0.01951321	0.00220312	8.85709434	8.21E-19	3.63E-18
Bacteroidales UCG-001	0.97101524	0.04805996	0.00431028	11.150077	7.15E-29	3.61E-28
Victivallaceae	1.63504024	0.04549642	0.00394805	11.523781	1.00E-30	5.90E-30
WCHB1 41 uncultured	0.63161674	0.02284092	0.00464949	4.91256231	8.99E-07	2.80E-06
Eggerthellaceae	0.42245702	0.00785087	0.00387652	2.02523987	0.04284274	0.09461105
Desulfovibrionaceae	1.33350841	0.02477596	0.00300461	8.24599331	1.64E-16	6.43E-16
Campylobacteraceae	0.36011159	0.02246802	0.00664923	3.37904159	0.00072739	0.0017931
Marinifilaceae	1.0795931	0.03259325	0.00274105	11.8908113	1.32E-32	8.75E-32
Dysgonomonadaceae	0.4240709	0.0274436	0.00589212	4.65768126	3.20E-06	9.42E-06
Defluviitaleaceae	1.20091909	0.0236732	0.00218957	10.8118247	3.03E-27	1.46E-26
Eubacteriaceae	0.28369233	0.02711129	0.00552608	4.90606036	9.29E-07	2.81E-06
gir aah93h0	0.31872427	0.01833041	0.00576371	3.18031613	0.00147115	0.00354412
Pirellulaceae	0.15741561	0.01399368	0.00662901	2.1109765	0.03477433	0.07842722

* Coefficients and significance values were calculated with DESeq2.

Table S3. Temperature (°C) and relative humidity (RH) the cattle farms during four-sampling phases.

	Farms									
	**1B**		**2D**		**4D**		**8B**		**9D**	
**Phase**	**°C**	**RH**	**°C**	**RH**	**°C**	**RH**	**°C**	**RH**	**°C**	**RH**
1	13.9	56.7	19.2	66.2	-	-	22.8	71.1	30.1	46.3
2	19.7	93.7	24.8	59.8	23.6	62.1	24.2	46.9	24.9	35.3
3	23.3	54.2	25.8	84.6	19.3	69.2	24.2	50.7	18.0	53.0
4	16.6	66.2	19.7	68.8	13.7	82.9	22.1	68.5	18.0	56.3
**Mean**	18.4	67.7	22.4	69.8	18.9	71.4	23.3	59.3	22.8	47.7
**St. dev.**	4.1	18.1	3.4	10.5	5.0	10.6	1.1	12.3	5.9	9.3

Table S4. Differentially abundant taxa identified between STEC shedders and non-shedders among dairy farms.

Taxa	Base Mean	log2 Fold Change	Lfc SE	Stat (Wald)	p-value	p-adj
Acidaminococcaceae	16.964	-0.565	0.136	-4.144	3.42E-05	6.27E-04
Actinobacteria	1.733	-0.126	0.346	-0.365	7.15E-01	9.74E-01
Alloprevotella	18.823	0.704	0.192	3.670	2.43E-04	3.05E-03
Anaerosporobacter	5.126	-1.363	0.340	-4.011	6.04E-05	1.33E-03
Anaerovorax	1.491	-0.598	0.252	-2.369	1.78E-02	8.26E-02
Bacilli	1.779	2.008	0.469	4.282	1.86E-05	5.38E-04
Bacteroidales RF16 group	49.233	-0.382	0.161	-2.374	1.76E-02	9.64E-02
Bacteroidales RF16 group uncultured	47.789	-0.368	0.159	-2.312	2.08E-02	8.30E-02
Bacteroidetes	805.395	0.086	0.068	1.265	2.06E-01	8.74E-01
Betaproteobacteriales	8.110	-1.287	0.340	-3.788	1.52E-04	8.20E-03
Butyrivibrio	2.007	-0.706	0.290	-2.437	1.48E-02	7.24E-02
Chloroflexi	0.005	-0.138	4.294	-0.032	9.74E-01	9.74E-01
Clostridia	1295.258	0.215	0.080	2.700	6.93E-03	6.70E-02
Clostridiales vadinBB60 group	12.878	-0.770	0.229	-3.366	7.64E-04	6.00E-03
Clostridiales vadinBB60 group uncultured	12.592	-0.779	0.225	-3.468	5.24E-04	5.50E-03
Cyanobacteria	11.310	-0.250	0.253	-0.987	3.24E-01	9.25E-01
Dependentiae	0.015	-0.148	4.294	-0.034	9.73E-01	9.74E-01
dgA 11 gut group	12.519	-0.666	0.230	-2.891	3.84E-03	2.39E-02
Elusimicrobia	0.121	-0.583	0.943	-0.618	5.37E-01	9.74E-01
EMP G18	1.703	-1.015	0.324	-3.137	1.71E-03	3.08E-02
Epsilonbacteraeota	0.387	0.387	0.650	0.596	5.51E-01	9.74E-01
Erysipelotrichaceae UCG 004	5.192	-0.768	0.236	-3.250	1.15E-03	9.23E-03
Euryarchaeota	0.103	-0.215	1.175	-0.183	8.55E-01	9.74E-01
F082	17.074	-0.777	0.264	-2.947	3.20E-03	2.20E-02
F082 uncultured	16.498	-0.741	0.258	-2.872	4.08E-03	2.39E-02
Family XIII AD3011 group	11.696	0.293	0.138	2.124	3.36E-02	9.87E-02
Fibrobacteres	1.117	-0.601	0.537	-1.118	2.63E-01	8.78E-01
Firmicutes	1374.505	0.270	0.085	3.178	1.48E-03	1.48E-02
Flavobacteriaceae	1.114	-0.778	0.338	-2.300	2.15E-02	9.84E-02
Fusobacteria	0.045	-0.140	3.951	-0.036	9.72E-01	9.74E-01
Gammaproteobacteria	30.401	-0.970	0.298	-3.255	1.13E-03	1.64E-02
Gastranaerophilales uncultured	11.109	-0.561	0.262	-2.144	3.20E-02	9.87E-02
Izimaplasmatales	4.561	-0.872	0.265	-3.287	1.01E-03	2.73E-02
Izimaplasmatales uncultured	4.574	-1.034	0.279	-3.699	2.16E-04	2.97E-03
Kingella	2.680	-1.985	0.599	-3.315	9.15E-04	8.05E-03
Kiritimatiellaeota	0.441	-0.352	0.544	-0.648	5.17E-01	9.74E-01
Lachnospiraceae uncultured	49.097	-0.255	0.117	-2.171	3.00E-02	9.87E-02
Lentisphaerae	1.809	-0.560	0.455	-1.231	2.18E-01	8.74E-01
Neisseriaceae	2.832	-2.102	0.606	-3.468	5.24E-04	5.33E-03
Oscillibacter	8.507	-0.586	0.170	-3.449	5.63E-04	5.50E-03
p 251 o5	6.588	-1.479	0.332	-4.453	8.45E-06	4.65E-04
p 251 o5 uncultured	6.394	-1.473	0.327	-4.501	6.76E-06	5.95E-04
Patescibacteria	0.171	-0.333	0.847	-0.393	6.94E-01	9.74E-01
Peptostreptococcaceae	3.878	1.096	0.259	4.225	2.38E-05	6.27E-04
Phascolarctobacterium	16.879	-0.551	0.135	-4.072	4.66E-05	1.33E-03
Planctomycetes	0.206	0.072	0.694	0.103	9.18E-01	9.74E-01
Proteobacteria	37.276	-0.772	0.209	-3.696	2.19E-04	4.38E-03
Rhodospirillales uncultured	7.588	-0.599	0.254	-2.362	1.82E-02	9.64E-02
Rikenellaceae	307.035	-0.232	0.099	-2.340	1.93E-02	9.64E-02
Rikenellaceae RC9 gut group	201.900	-0.307	0.100	-3.063	2.19E-03	1.49E-02
Romboutsia	3.706	1.039	0.268	3.882	1.04E-04	1.83E-03
Roseburia	17.073	-0.971	0.226	-4.302	1.69E-05	7.45E-04
Ruminobacter	6.761	-1.070	0.434	-2.467	1.36E-02	7.05E-02
Ruminococcaceae UCG 005	292.663	0.220	0.087	2.520	1.17E-02	6.46E-02
Ruminococcaceae UCG 010	201.363	-0.366	0.172	-2.131	3.31E-02	9.87E-02
Ruminococcaceae UCG 011	1.814	0.761	0.244	3.116	1.83E-03	1.34E-02
Ruminococcus 1	19.058	0.308	0.142	2.172	2.98E-02	9.87E-02
Ruminococcus 2	4.029	-0.521	0.240	-2.174	2.97E-02	9.87E-02

Table S4. Continued…

Taxa	Base Mean	log2 Fold Change	Lfc SE	Stat (Wald)	p-value	p-adj
Spirochaetes	22.492	0.202	0.235	0.858	3.91E-01	9.74E-01
Synergistetes	0.009	-0.162	4.294	-0.038	9.70E-01	9.74E-01
Tenericutes	40.104	0.011	0.108	0.106	9.16E-01	9.74E-01
Tyzzerella 4	10.987	0.404	0.174	2.319	2.04E-02	8.30E-02
Veillonellaceae	6.441	1.191	0.346	3.440	5.82E-04	5.33E-03
Verrucomicrobia	3.755	0.691	0.314	2.203	2.76E-02	1.84E-01
Eubacterium coprostanoligenes group	95.526	0.227	0.098	2.323	2.02E-02	8.30E-02
Eubacterium ruminantium group	2.788	0.578	0.262	2.208	2.72E-02	9.87E-02

Table S5. Taxa correlated with factors associated with STEC carriage.

Risk factor	Taxa	Base Mean	log2 Fold Change	Lfc SE	Stat (Wald)	p-value	p-adj
DIM	*Acetitomaculum*	1.090	0.003	0.001	2.447	1.44E-02	9.94E-02
DIM	*Actinobacteria*	3.135	0.004	0.001	3.432	5.99E-04	5.99E-03
DIM	*Anaerosporobacter*	5.185	0.003	0.001	3.159	1.58E-03	4.15E-02
DIM	*Anaerovibrio*	5.509	-0.005	0.001	-4.567	4.94E-06	6.47E-04
DIM	*Cellulosilyticum*	1.657	-0.003	0.001	-2.631	8.52E-03	7.44E-02
DIM	*Coprococcus 3*	10.675	-0.001	0.001	-2.546	1.09E-02	8.34E-02
DIM	*Enterobacteriaceae*	3.542	-0.005	0.002	-3.417	6.34E-04	3.04E-02
DIM	*Escherichia Shigella*	4.099	-0.006	0.002	-3.382	7.19E-04	2.36E-02
DIM	*hoa5 07d05 gut group*	1.354	0.009	0.002	3.844	1.21E-04	6.85E-03
DIM	*Kingella*	4.855	0.004	0.002	2.528	1.15E-02	8.34E-02
DIM	*Mollicutes RF39 uncultured*	28.987	-0.001	0.000	-2.792	5.23E-03	6.86E-02
DIM	*Prevotellaceae UCG 003*	65.196	-0.001	0.000	-3.780	1.57E-04	6.85E-03
DIM	*Ruminiclostridium*	3.758	0.002	0.001	2.667	7.65E-03	7.44E-02
DIM	*Ruminiclostridium 1*	2.112	0.002	0.001	2.738	6.19E-03	7.37E-02
DIM	*Ruminiclostridium 6*	1.690	-0.002	0.001	-2.540	1.11E-02	8.34E-02
DIM	*Ruminococcaceae*	878.919	0.001	0.000	3.785	1.54E-04	1.48E-02
DIM	*Ruminococcaceae UCG 005*	291.274	0.001	0.000	2.850	4.37E-03	6.36E-02
DIM	*Ruminococcaceae UCG 009*	20.770	0.001	0.000	2.679	7.39E-03	7.44E-02
DIM	*Ruminococcaceae uncultured*	53.067	0.001	0.000	2.885	3.92E-03	6.36E-02
DIM	*Tenericutes*	39.895	-0.001	0.000	-3.494	4.75E-04	5.99E-03
DIM	*Turicibacter*	1.191	-0.003	0.001	-3.039	2.37E-03	5.18E-02
DIM	*Veillonellaceae*	6.451	-0.003	0.001	-3.125	1.78E-03	5.70E-02
DIM	*Veillonellaceae uncultured*	0.987	-0.003	0.001	-2.653	7.99E-03	7.44E-02
DIM	*Eubacterium ruminantium group*	2.802	-0.002	0.001	-2.864	4.18E-03	6.36E-02
Lactations	*Kingella*	4.855	0.610	0.167	3.644	2.69E-04	4.06E-02
Lactations	*Lentisphaerae*	1.779	-0.392	0.125	-3.135	1.72E-03	3.43E-02
Lactations	*Neisseriaceae*	5.068	0.631	0.169	3.738	1.86E-04	1.87E-02
Lactations	*Ruminococcaceae UCG 004*	2.279	-0.226	0.066	-3.409	6.52E-04	5.04E-02
Lactations	*Ruminococcaceae UCG 011*	1.809	-0.247	0.076	-3.267	1.09E-03	6.56E-02
Lactations	*Victivallaceae uncultured*	1.182	-0.530	0.143	-3.706	2.11E-04	4.06E-02
Lactations	*Eubacterium oxidoreducens group*	7.138	-0.177	0.052	-3.403	6.67E-04	5.04E-02
Temp. ave. 5 days	*Acetitomaculum*	0.589	0.060	0.026	2.342	1.92E-02	3.54E-02
Temp. ave. 5 days	*Acidaminococcaceae*	21.088	-0.073	0.007	-10.709	9.28E-27	7.66E-26
Temp. ave. 5 days	*Agathobacter*	5.579	-0.058	0.016	-3.659	2.53E-04	5.68E-04
Temp. ave. 5 days	*Akkermansia*	3.372	0.155	0.021	7.309	2.68E-13	1.11E-12
Temp. ave. 5 days	*Akkermansiaceae*	2.829	0.124	0.020	6.274	3.52E-10	1.01E-09
Temp. ave. 5 days	*Alistipes*	109.863	0.190	0.013	15.161	6.45E-52	2.87E-50
Temp. ave. 5 days	*Alloprevotella*	36.572	-0.082	0.011	-7.130	1.00E-12	4.03E-12
Temp. ave. 5 days	*Anaerofustis*	0.350	0.173	0.040	4.290	1.79E-05	4.27E-05
Temp. ave. 5 days	*Anaeroplasma*	3.288	-0.046	0.016	-2.781	5.42E-03	1.12E-02
Temp. ave. 5 days	*Anaeroplasmataceae*	3.442	-0.085	0.016	-5.210	1.89E-07	4.16E-07
Temp. ave. 5 days	*Anaerosporobacter*	3.314	-0.072	0.016	-4.544	5.51E-06	1.39E-05
Temp. ave. 5 days	*Anaerostipes*	0.385	-0.089	0.037	-2.370	1.78E-02	3.33E-02
Temp. ave. 5 days	*Anaerovorax*	1.744	0.084	0.015	5.414	6.17E-08	1.81E-07
Temp. ave. 5 days	*Angelakisella*	0.488	0.060	0.025	2.466	1.36E-02	2.63E-02
Temp. ave. 5 days	*Bacteroidaceae*	158.040	0.041	0.008	5.264	1.41E-07	3.21E-07
Temp. ave. 5 days	*Bacteroidales RF16 group*	39.216	0.108	0.013	8.197	2.47E-16	1.02E-15
Temp. ave. 5 days	*Bacteroidales RF16 group uncultured*	45.003	0.136	0.015	9.296	1.45E-20	9.29E-20
Temp. ave. 5 days	*Bacteroidales UCG 001*	0.969	0.263	0.033	7.860	3.85E-15	1.49E-14
Temp. ave. 5 days	*Bacteroidales UCG 001 uncultured*	1.414	0.327	0.036	9.182	4.23E-20	2.51E-19
Temp. ave. 5 days	*Bacteroidales uncultured*	37.465	0.137	0.015	9.228	2.76E-20	1.30E-19
Temp. ave. 5 days	*Bacteroidales uncultured uncultured*	48.553	0.177	0.017	10.592	3.26E-26	2.70E-25
Temp. ave. 5 days	*Bacteroides*	172.942	0.081	0.009	8.750	2.13E-18	1.10E-17
Temp. ave. 5 days	*Bacteroidetes*	815.667	-0.027	0.003	-10.258	1.09E-24	1.09E-23
Temp. ave. 5 days	*Barnesiellaceae*	3.845	0.088	0.014	6.140	8.26E-10	2.18E-09
Temp. ave. 5 days	*Barnesiellaceae uncultured*	4.623	0.126	0.016	7.981	1.45E-15	7.07E-15
Temp. ave. 5 days	*Blautia*	2.917	-0.054	0.012	-4.320	1.56E-05	3.77E-05
Temp. ave. 5 days	*Breznakia*	0.515	0.057	0.022	2.573	1.01E-02	2.01E-02

Table S5. Continued…

Risk factor	Taxa	Base Mean	log2 Fold Change	Lfc SE	Stat (Wald)	p-value	p-adj
Temp. ave. 5 days	*Burkholderiaceae*	3.976	-0.124	0.013	-9.305	1.34E-20	6.82E-20
Temp. ave. 5 days	*Butyrivibrio*	2.268	-0.097	0.015	-6.493	8.42E-11	3.08E-10
Temp. ave. 5 days	*Campylobacter*	0.496	0.125	0.045	2.765	5.68E-03	1.16E-02
Temp. ave. 5 days	*Campylobacteraceae*	0.361	0.083	0.044	1.878	6.04E-02	9.73E-02
Temp. ave. 5 days	*Candidatus Soleaferrea*	8.859	0.106	0.012	8.614	7.08E-18	3.60E-17
Temp. ave. 5 days	*Candidatus Stoquefichus*	0.565	0.153	0.029	5.343	9.14E-08	2.65E-07
Temp. ave. 5 days	*Caproiciproducens*	3.157	0.153	0.021	7.251	4.14E-13	1.69E-12
Temp. ave. 5 days	*Christensenellaceae*	58.524	0.092	0.009	10.064	8.00E-24	4.40E-23
Temp. ave. 5 days	*Christensenellaceae R 7 group*	65.228	0.134	0.011	12.767	2.51E-37	4.80E-36
Temp. ave. 5 days	*Christensenellaceae uncultured*	1.045	0.161	0.021	7.670	1.72E-14	7.40E-14
Temp. ave. 5 days	*Clostridiaceae 1*	10.278	-0.098	0.016	-6.157	7.40E-10	2.03E-09
Temp. ave. 5 days	*Clostridiales vadinBB60 group uncultured*	17.824	0.052	0.014	3.774	1.61E-04	3.67E-04
Temp. ave. 5 days	*Clostridium sensu stricto 1*	8.921	-0.086	0.015	-5.645	1.65E-08	5.08E-08
Temp. ave. 5 days	*Clostridium sensu stricto 6*	0.218	0.192	0.068	2.849	4.39E-03	9.11E-03
Temp. ave. 5 days	*Coprobacillus*	0.657	0.147	0.025	5.934	2.96E-09	9.46E-09
Temp. ave. 5 days	*Coprococcus 3*	8.955	0.077	0.011	6.808	9.88E-12	3.67E-11
Temp. ave. 5 days	*Defluviitaleaceae*	1.201	0.108	0.015	7.402	1.34E-13	4.91E-13
Temp. ave. 5 days	*Defluviitaleaceae UCG 011*	1.394	0.150	0.016	9.208	3.33E-20	2.02E-19
Temp. ave. 5 days	*Desulfovibrionaceae*	1.345	0.079	0.020	3.846	1.20E-04	2.47E-04
Temp. ave. 5 days	*dgA 11 gut group*	15.168	0.251	0.020	12.338	5.67E-35	8.83E-34
Temp. ave. 5 days	*Dielma*	1.601	0.207	0.018	11.579	5.26E-31	5.95E-30
Temp. ave. 5 days	*Dorea*	1.557	0.140	0.015	9.211	3.22E-20	2.01E-19
Temp. ave. 5 days	*Dysgonomonadaceae*	0.429	0.085	0.039	2.145	3.20E-02	5.41E-02
Temp. ave. 5 days	*Dysgonomonadaceae uncultured*	0.574	0.129	0.041	3.151	1.62E-03	3.40E-03
Temp. ave. 5 days	*Eisenbergiella*	0.600	0.113	0.022	5.089	3.61E-07	9.76E-07
Temp. ave. 5 days	*EMP G18 uncultured*	0.699	-0.219	0.025	-8.912	5.01E-19	2.21E-18
Temp. ave. 5 days	*EMP G18 uncultured uncultured*	0.579	-0.171	0.024	-7.026	2.13E-12	8.42E-12
Temp. ave. 5 days	*Enterobacteriaceae*	3.896	-0.278	0.026	-10.904	1.10E-27	1.04E-26
Temp. ave. 5 days	*Enterorhabdus*	0.263	0.078	0.032	2.403	1.62E-02	3.06E-02
Temp. ave. 5 days	*Erysipelatoclostridium*	1.148	0.134	0.019	6.993	2.68E-12	1.04E-11
Temp. ave. 5 days	*Erysipelotrichaceae*	25.112	-0.039	0.007	-5.951	2.66E-09	6.76E-09
Temp. ave. 5 days	*Erysipelotrichaceae UCG 004*	7.657	-0.118	0.012	-9.503	2.03E-21	1.41E-20
Temp. ave. 5 days	*Erysipelotrichaceae uncultured*	4.261	0.061	0.012	5.000	5.74E-07	1.54E-06
Temp. ave. 5 days	*Escherichia Shigella*	3.707	-0.251	0.026	-9.621	6.52E-22	4.64E-21
Temp. ave. 5 days	*Eubacteriaceae*	0.282	0.125	0.039	3.205	1.35E-03	2.62E-03
Temp. ave. 5 days	*F082*	16.219	0.279	0.021	13.111	2.83E-39	6.23E-38
Temp. ave. 5 days	*F082 uncultured*	22.149	0.307	0.023	13.170	1.30E-39	4.04E-38
Temp. ave. 5 days	*Faecalibacterium*	1.676	-0.294	0.024	-12.485	9.01E-36	1.50E-34
Temp. ave. 5 days	*Faecalitalea*	0.722	0.089	0.024	3.764	1.67E-04	3.79E-04
Temp. ave. 5 days	*Family XIII*	18.016	0.084	0.008	10.242	1.29E-24	7.72E-24
Temp. ave. 5 days	*Family XIII AD3011 group*	14.182	0.120	0.010	11.735	8.45E-32	1.00E-30
Temp. ave. 5 days	*Family XIII UCG 001*	1.008	0.147	0.019	7.822	5.18E-15	2.39E-14
Temp. ave. 5 days	*Fibrobacter*	2.692	-0.065	0.032	-2.025	4.29E-02	7.68E-02
Temp. ave. 5 days	*Fibrobacteraceae*	2.368	-0.092	0.031	-3.006	2.65E-03	4.86E-03
Temp. ave. 5 days	*Fibrobacteres*	2.227	-0.113	0.030	-3.738	1.85E-04	5.30E-04
Temp. ave. 5 days	*Firmicutes*	1213.811	0.024	0.003	7.693	1.43E-14	9.56E-14
Temp. ave. 5 days	*Fournierella*	0.385	-0.183	0.042	-4.386	1.15E-05	2.82E-05
Temp. ave. 5 days	*Gastranaerophilales uncultured*	11.682	0.025	0.013	1.936	5.28E-02	8.72E-02
Temp. ave. 5 days	*Gastranaerophilales uncultured uncultured*	14.264	0.065	0.015	4.439	9.03E-06	2.23E-05
Temp. ave. 5 days	*GCA 900066225*	0.482	0.057	0.022	2.597	9.40E-03	1.89E-02
Temp. ave. 5 days	*gir aah93h0 uncultured*	0.432	0.099	0.039	2.526	1.15E-02	2.26E-02
Temp. ave. 5 days	*GWE2 31 10*	0.908	0.152	0.036	4.258	2.06E-05	4.89E-05
Temp. ave. 5 days	*hoa5 07d05 gut group*	3.627	-0.319	0.032	-10.065	7.85E-24	5.92E-23
Temp. ave. 5 days	*Hydrogenoanaerobacterium*	0.533	0.136	0.029	4.736	2.18E-06	5.53E-06
Temp. ave. 5 days	*Intestinimonas*	0.252	0.117	0.050	2.351	1.87E-02	3.48E-02
Temp. ave. 5 days	*Izimaplasmatales uncultured*	4.620	0.057	0.015	3.772	1.62E-04	3.24E-04
Temp. ave. 5 days	*Izimaplasmatales uncultured uncultured*	5.922	0.091	0.017	5.286	1.25E-07	3.50E-07
Temp. ave. 5 days	*Kingella*	3.801	-0.156	0.041	-3.815	1.36E-04	3.14E-04

Table S5. Continued…

Risk factor	Taxa	Base Mean	log2 Fold Change	Lfc SE	Stat (Wald)	p-value	p-adj
Temp. ave. 5 days	*Lachnoclostridium*	2.147	-0.069	0.014	-4.900	9.58E-07	2.48E-06
Temp. ave. 5 days	*Lachnoclostridium 10*	1.954	0.122	0.018	6.886	5.72E-12	2.19E-11
Temp. ave. 5 days	*Lachnospira*	0.378	0.120	0.027	4.483	7.35E-06	1.83E-05
Temp. ave. 5 days	*Lachnospiraceae FCS020 group*	7.043	0.087	0.011	7.911	2.55E-15	1.22E-14
Temp. ave. 5 days	*Lachnospiraceae NC2004 group*	0.196	0.100	0.039	2.546	1.09E-02	2.15E-02
Temp. ave. 5 days	*Lachnospiraceae NK3A20 group*	0.815	0.105	0.021	4.966	6.85E-07	1.79E-06
Temp. ave. 5 days	*Lachnospiraceae NK4A136 group*	30.785	0.084	0.008	11.208	3.72E-29	3.20E-28
Temp. ave. 5 days	*Lachnospiraceae UCG 001*	2.267	0.041	0.012	3.494	4.75E-04	1.03E-03
Temp. ave. 5 days	*Lachnospiraceae UCG 008*	0.807	0.122	0.018	6.866	6.58E-12	2.48E-11
Temp. ave. 5 days	*Lachnospiraceae UCG 010*	0.359	0.090	0.025	3.604	3.13E-04	6.84E-04
Temp. ave. 5 days	*Lentisphaerae*	1.494	0.118	0.024	4.853	1.22E-06	4.06E-06
Temp. ave. 5 days	*Lysinibacillus*	3.163	0.333	0.044	7.569	3.76E-14	1.59E-13
Temp. ave. 5 days	*Mailhella*	1.720	0.119	0.022	5.303	1.14E-07	3.22E-07
Temp. ave. 5 days	*Marinifilaceae*	1.084	0.133	0.019	7.179	7.03E-13	2.44E-12
Temp. ave. 5 days	*Marvinbryantia*	0.676	0.035	0.018	1.913	5.57E-02	9.84E-02
Temp. ave. 5 days	*Mogibacterium*	0.451	0.153	0.025	6.026	1.68E-09	5.67E-09
Temp. ave. 5 days	*Mollicutes RF39 uncultured*	28.057	0.024	0.009	2.730	6.33E-03	1.13E-02
Temp. ave. 5 days	*Mollicutes RF39 uncultured uncultured*	30.091	0.056	0.010	5.630	1.80E-08	5.48E-08
Temp. ave. 5 days	*Muribaculaceae*	82.089	-0.186	0.010	-18.328	4.94E-75	3.26E-73
Temp. ave. 5 days	*Muribaculaceae uncultured*	76.161	-0.156	0.010	-15.156	6.90E-52	2.87E-50
Temp. ave. 5 days	*Muribaculum*	0.212	-0.102	0.042	-2.423	1.54E-02	2.92E-02
Temp. ave. 5 days	*Negativibacillus*	5.267	0.143	0.012	11.494	1.41E-30	1.53E-29
Temp. ave. 5 days	*Neisseriaceae*	4.335	-0.196	0.041	-4.768	1.86E-06	3.96E-06
Temp. ave. 5 days	*Odoribacter*	1.244	0.198	0.021	9.368	7.39E-21	4.84E-20
Temp. ave. 5 days	*Oscillibacter*	11.406	-0.073	0.009	-7.775	7.56E-15	3.42E-14
Temp. ave. 5 days	*Oscillospira*	0.428	-0.147	0.029	-5.138	2.77E-07	7.58E-07
Temp. ave. 5 days	*p 1088 a5 gut group*	0.222	0.102	0.045	2.299	2.15E-02	3.91E-02
Temp. ave. 5 days	*p 251 o5 uncultured*	7.801	0.060	0.022	2.735	6.24E-03	1.26E-02
Temp. ave. 5 days	*p 2534 18B5 gut group*	11.434	0.324	0.027	12.144	6.15E-34	6.76E-33
Temp. ave. 5 days	*p 2534 18B5 gut group uncultured*	15.088	0.348	0.028	12.279	1.17E-34	1.71E-33
Temp. ave. 5 days	*Paeniclostridium*	1.292	0.071	0.029	2.498	1.25E-02	2.43E-02
Temp. ave. 5 days	*Paludibacter*	0.555	0.133	0.041	3.251	1.15E-03	2.43E-03
Temp. ave. 5 days	*Paludibacteraceae*	5.829	0.183	0.017	10.595	3.13E-26	2.29E-25
Temp. ave. 5 days	*Paludibacteraceae uncultured*	6.698	0.240	0.019	12.580	2.71E-36	4.81E-35
Temp. ave. 5 days	*Papillibacter*	1.281	0.205	0.022	9.454	3.27E-21	2.20E-20
Temp. ave. 5 days	*Parabacteroides*	10.695	-0.215	0.016	-13.027	8.61E-39	2.38E-37
Temp. ave. 5 days	*Parasutterella*	1.714	0.119	0.019	6.212	5.24E-10	1.79E-09
Temp. ave. 5 days	*Peptococcaceae*	9.032	0.046	0.009	5.331	9.77E-08	2.30E-07
Temp. ave. 5 days	*Peptococcaceae uncultured*	10.223	0.089	0.010	8.887	6.30E-19	3.48E-18
Temp. ave. 5 days	*Phascolarctobacterium*	21.559	-0.039	0.008	-4.863	1.16E-06	2.97E-06
Temp. ave. 5 days	*Planococcaceae*	3.612	0.294	0.041	7.109	1.17E-12	3.86E-12
Temp. ave. 5 days	*Prevotella 2*	12.415	-0.635	0.037	-17.189	3.23E-66	4.02E-64
Temp. ave. 5 days	*Prevotella 9*	25.457	-0.442	0.034	-12.852	8.38E-38	1.90E-36
Temp. ave. 5 days	*Prevotellaceae*	215.494	-0.080	0.006	-12.926	3.22E-38	5.32E-37
Temp. ave. 5 days	*Prevotellaceae Ga6A1 group*	0.675	0.107	0.028	3.869	1.09E-04	2.54E-04
Temp. ave. 5 days	*Prevotellaceae NK3B31 group*	3.745	-0.243	0.041	-5.942	2.81E-09	9.09E-09
Temp. ave. 5 days	*Prevotellaceae UCG 001*	14.031	-0.077	0.012	-6.343	2.26E-10	7.93E-10
Temp. ave. 5 days	*Prevotellaceae UCG 003*	72.565	0.089	0.010	8.760	1.95E-18	1.03E-17
Temp. ave. 5 days	*Prevotellaceae UCG 004*	29.292	0.247	0.017	14.828	9.69E-50	3.45E-48
Temp. ave. 5 days	*Prevotellaceae uncultured*	13.029	-0.435	0.034	-12.972	1.77E-38	4.40E-37
Temp. ave. 5 days	*Proteobacteria*	45.934	-0.134	0.012	-11.193	4.40E-29	8.79E-28
Temp. ave. 5 days	*Rhodospirillales uncultured*	7.810	0.040	0.013	3.071	2.13E-03	4.02E-03
Temp. ave. 5 days	*Rikenellaceae*	280.580	0.079	0.008	10.351	4.16E-25	2.74E-24
Temp. ave. 5 days	*Rikenellaceae RC9 gut group*	195.183	0.094	0.009	9.998	1.55E-23	1.14E-22
Temp. ave. 5 days	*Romboutsia*	3.852	0.033	0.014	2.331	1.98E-02	3.62E-02
Temp. ave. 5 days	*Roseburia*	12.998	0.020	0.010	1.971	4.88E-02	8.68E-02

Risk factor	Taxa	Base Mean	log2 Fold Change	Lfc SE	Stat (Wald)	p-value	p-adj
Temp. ave. 5 days	*Ruminiclostridium*	5.484	0.220	0.018	12.235	2.02E-34	2.80E-33
Temp. ave. 5 days	*Ruminiclostridium 1*	2.571	0.209	0.020	10.580	3.71E-26	2.98E-25
Temp. ave. 5 days	*Ruminiclostridium 5*	1.505	0.115	0.015	7.858	3.89E-15	1.83E-14
Temp. ave. 5 days	*Ruminiclostridium 6*	1.453	0.069	0.019	3.619	2.95E-04	6.51E-04
Temp. ave. 5 days	*Ruminiclostridium 9*	4.310	0.045	0.014	3.276	1.05E-03	2.24E-03
Temp. ave. 5 days	*Ruminobacter*	8.235	-0.135	0.023	-5.850	4.91E-09	1.55E-08
Temp. ave. 5 days	*Ruminococcaceae*	925.563	0.075	0.006	12.879	5.90E-38	7.79E-37
Temp. ave. 5 days	*Ruminococcaceae NK4A214 group*	15.240	0.213	0.012	17.462	2.80E-68	6.96E-66
Temp. ave. 5 days	*Ruminococcaceae UCG 002*	14.349	0.125	0.014	9.070	1.19E-19	6.89E-19
Temp. ave. 5 days	*Ruminococcaceae UCG 004*	2.800	0.158	0.014	11.283	1.59E-29	1.42E-28
Temp. ave. 5 days	*Ruminococcaceae UCG 005*	373.131	0.148	0.009	16.907	3.98E-64	3.30E-62
Temp. ave. 5 days	*Ruminococcaceae UCG 008*	0.559	0.104	0.019	5.479	4.27E-08	1.27E-07
Temp. ave. 5 days	*Ruminococcaceae UCG 009*	23.939	0.151	0.013	11.823	2.97E-32	3.70E-31
Temp. ave. 5 days	*Ruminococcaceae UCG 010*	276.254	0.150	0.013	11.455	2.21E-30	2.20E-29
Temp. ave. 5 days	*Ruminococcaceae UCG 011*	3.298	0.110	0.017	6.343	2.25E-10	7.93E-10
Temp. ave. 5 days	*Ruminococcaceae UCG 013*	80.548	0.061	0.010	5.952	2.64E-09	8.77E-09
Temp. ave. 5 days	*Ruminococcaceae UCG 014*	33.964	0.048	0.010	4.969	6.73E-07	1.78E-06
Temp. ave. 5 days	*Ruminococcaceae uncultured*	65.302	0.164	0.010	16.411	1.60E-60	9.96E-59
Temp. ave. 5 days	*Ruminococcus 1*	17.235	0.093	0.008	11.305	1.24E-29	1.14E-28
Temp. ave. 5 days	*Ruminococcus 2*	3.113	0.112	0.015	7.675	1.65E-14	7.23E-14
Temp. ave. 5 days	*Saccharofermentans*	0.711	0.171	0.032	5.304	1.14E-07	3.22E-07
Temp. ave. 5 days	*Solibacillus*	1.616	0.470	0.074	6.321	2.60E-10	9.01E-10
Temp. ave. 5 days	*Sphaerochaeta*	0.534	0.143	0.026	5.583	2.36E-08	7.08E-08
Temp. ave. 5 days	*Spirochaetaceae*	38.677	-0.087	0.015	-5.738	9.57E-09	2.34E-08
Temp. ave. 5 days	*Spirochaetes*	39.978	-0.106	0.015	-6.891	5.55E-12	2.78E-11
Temp. ave. 5 days	*Subdoligranulum*	1.680	-0.256	0.022	-11.442	2.58E-30	2.47E-29
Temp. ave. 5 days	*Succinivibrio*	6.431	-0.132	0.025	-5.214	1.84E-07	5.10E-07
Temp. ave. 5 days	*Succinivibrionaceae*	19.125	-0.145	0.021	-6.811	9.67E-12	2.90E-11
Temp. ave. 5 days	*Sutterella*	1.566	-0.290	0.025	-11.484	1.59E-30	1.65E-29
Temp. ave. 5 days	*Tannerellaceae*	11.853	-0.245	0.016	-15.034	4.39E-51	1.45E-49
Temp. ave. 5 days	*Treponema 2*	36.316	-0.067	0.016	-4.240	2.23E-05	5.24E-05
Temp. ave. 5 days	*Turicibacter*	3.409	-0.151	0.020	-7.720	1.17E-14	5.18E-14
Temp. ave. 5 days	*Tyzzerella 4*	11.143	0.057	0.010	5.743	9.31E-09	2.90E-08
Temp. ave. 5 days	*Verrucomicrobia*	2.847	0.116	0.019	6.061	1.35E-09	5.42E-09
Temp. ave. 5 days	*Victivallaceae*	1.662	0.200	0.028	7.059	1.67E-12	5.26E-12
Temp. ave. 5 days	*Victivallaceae uncultured*	1.739	0.297	0.033	8.977	2.79E-19	1.58E-18
Temp. ave. 5 days	*WCHB1 41 uncultured*	0.635	0.076	0.032	2.396	1.66E-02	2.88E-02
Temp. ave. 5 days	*WCHB1 41 uncultured uncultured*	0.883	0.123	0.034	3.628	2.86E-04	6.35E-04
Temp. ave. 5 days	*Anaerorhabdus furcosa group*	3.852	0.162	0.013	12.808	1.49E-37	3.09E-36
Temp. ave. 5 days	*Eubacterium brachy group*	1.357	0.138	0.017	8.144	3.83E-16	1.91E-15
Temp. ave. 5 days	*Eubacterium coprostanoligenes group*	124.788	0.088	0.010	8.801	1.36E-18	7.35E-18
Temp. ave. 5 days	*Eubacterium eligens group*	0.384	0.170	0.029	5.945	2.77E-09	9.07E-09
Temp. ave. 5 days	*Eubacterium nodatum group*	1.953	0.194	0.016	11.889	1.34E-32	1.76E-31
Temp. ave. 5 days	*Eubacterium oxidoreducens group*	6.591	0.106	0.010	10.217	1.67E-24	1.30E-23
Temp. ave. 5 days	*Eubacterium ruminantium group*	2.313	0.060	0.018	3.370	7.53E-04	1.62E-03
Temp. ave. 5 days	*Eubacterium ventriosum group*	0.274	-0.074	0.034	-2.160	3.08E-02	5.55E-02
Temp. ave. 5 days	*Ruminococcus torques group*	1.263	-0.111	0.017	-6.423	1.34E-10	4.82E-10
Temp. ave. 5 days	*X28 4*	0.345	0.076	0.031	2.464	1.37E-02	2.63E-02
